# Structure and mechanism of the HSV-1 origin-binding protein UL9

**DOI:** 10.1128/jvi.00271-26

**Published:** 2026-07-10

**Authors:** Cuiqing Huang, Haiqiang Wu, Jinmiao Song, Xinzheng Zhang, Jun Ma

**Affiliations:** 1 State Key Laboratory of Biomacromolecules, Institute of Biophysics, Chinese Academy of Sciences 53015, Beijing, China; 2 University of Chinese Academy of Sciences 74519, Beijing, China; 3 Institute of Infectious Diseases, Shenzhen Bay Laboratory 551667, Shenzhen, China; 4 College of Veterinary Medicine, Anhui Agricultural University 12486, Hefei, China; University of Virginia, Charlottesville, Virginia, USA

**Keywords:** herpesvirus, DNA replication machinery, origin-binding protein, UL9, helicase superfamily, ssDNA-binding protein, ICP8

## Abstract

The herpesvirus DNA replication machinery comprises a battery of viral enzymes that orchestrate viral genome synthesis. In herpes simplex virus type 1 (HSV-1), the machinery consists of seven essential components, including the origin-binding protein UL9, the single-stranded DNA (ssDNA)-binding protein ICP8, the heterodimeric DNA polymerase complex UL30–UL42, and the heterotrimeric helicase–primase complex UL5–UL8–UL52. UL9, a superfamily 2 (SF2) helicase, functions as a dimer that specifically recognizes replication origins and unwinds duplex DNA to initiate replication. Furthermore, UL9 recruits the replication machinery through interactions with viral components and engages cellular proteins that regulate its function. However, the molecular mechanisms underlying the multifunctionality of UL9 remain incompletely understood due to the lack of structural information. Here, we present cryo-electron microscopy structures of UL9 in both apo and DNA-bound states. Together with biochemical and enzymatic assays, we elucidate the molecular basis of UL9 dimerization, origin recognition and allosteric regulation by ICP8.

**IMPORTANCE:**

Herpes simplex virus 1 (HSV-1) is a widespread virus that causes lifelong infections, leading to periodic outbreaks ranging from common cold sores to life-threatening encephalitis, and no current treatment can eradicate the dormant virus. To multiply, HSV-1 relies on a protein-based molecular machine to replicate its genome, where the unwinding of double-stranded DNA at specific replication origins is coordinated by the viral origin-binding protein UL9. Here, we present the high-resolution structures of UL9, both alone and bound to DNA, revealing how it forms a stable homodimer to grab onto the origin. Combined with precise biochemical experiments, we further show how UL9 collaborates with another viral helper protein, ICP8, to unwind DNA efficiently. These discoveries solve a long-standing puzzle in herpesvirus biology and offer a vital structural blueprint for designing new antiviral drugs that can block viral replication at its very earliest stage.

## INTRODUCTION

The *Herpesviridae* family comprises a large group of enveloped viruses with relatively large, complex double-stranded DNA genomes. To date, over 100 herpesviruses with a wide range of vertebrate hosts have been recognized and classified into three subfamilies (α-, β-, and γ-herpesviruses) based on their biological properties and tissue tropism. Notably, nine herpesviruses are pathogenic in humans and cause numerous human diseases; these include α-herpesviruses ‌(herpes simplex virus types 1 and 2 [HSV-1, HSV-2],‌ ‌varicella-zoster virus [VZV]‌); β-herpesviruses ‌(human cytomegalovirus [HCMV], human herpesvirus 6A/B [HHV-6A, HHV-6B]‌, ‌human herpesvirus 7 [HHV-7]‌); γ-herpesviruses ‌(Epstein-Barr virus [EBV]‌ and ‌Kaposi’s sarcoma-associated herpesvirus [KSHV/HHV-8]‌) (1). Over 90% of the global population is seropositive for one or more of these viruses. Herpesviruses establish lifelong latency in infected persons and periodically reactivate during episodes of immunosuppression or physiological stress, with no therapeutic approaches currently available to achieve complete viral eradication (2). To date, medical interventions for herpesvirus infections remain limited. Approved prophylactic options are restricted to VZV, including the Shingrix and Zostavax vaccines (3). Therapeutic small-molecule antivirals primarily target specific herpesviruses, with guanosine analogs and helicase-primase inhibitors—such as Acyclovir, Valacyclovir, Famciclovir, and Pritelivir—effective utilized against HSV-1 and VZV (4), and Maribavir deployed clinically for refractory HCMV infections (5). For other members of the *Herpesviridae* family, particularly oncogenic pathogens EBV and KSHV, effective specific antiviral drugs or vaccines remain unavailable. Furthermore, current long-term antiviral therapies carry significant risks of adverse side effects and the emergence of drug resistance (4, 6, 7). Consequently, the development of novel, highly specific therapeutic strategies against herpesviruses is essential, with viral DNA replication representing a particularly promising target for drug discovery (8, 9).

Herpesvirus DNA replication occurs in the nucleus of the host cell, carried out by a sophisticated multienzyme DNA replication machinery (10–12). In HSV-1, this machinery comprises seven core components, including the replication origin-binding protein (OBP) UL9, single-stranded DNA (ssDNA)-binding protein ICP8, the heterodimeric DNA polymerase complex UL30/UL42, and the heterotrimeric helicase–primase complex UL5/UL8/UL52. The DNA polymerase complex possesses two functionally distinct subunits: the catalytic subunit UL30 is a B-family DNA polymerase harboring both 5′–3′ polymerase activity and 3′–5′ proofreading exonuclease activity, and the processivity factor UL42 helps polymerase maintain high processivity. Within the helicase–primase complex, the helicase subunit UL5 is responsible for double-stranded DNA (dsDNA) unwinding during elongation, the primase subunit UL52 synthesizes short RNA primers for DNA polymerase, and the primase–helicase associated factor UL8 coordinates the activities of UL5 and UL52. The ssDNA-binding protein ICP8 is a multifunctional protein that prefers to bind ssDNA in a non-sequence-specific and cooperative manner while preventing it from nuclease degradation and annealing (11, 13). Additionally, ICP8 stimulates the helicase activity of UL9, modulates DNA polymerase processivity, and regulates helicase–primase complex function through direct interactions with UL9, UL42, and UL8 (10–12), respectively. The origin-binding protein UL9 mainly orchestrates replication initiation through sequence-specific recognition of origin sites and subsequently unwinds the genomic DNA with the help of ICP8 to initiate DNA replication (10–12, 14–22). Furthermore, UL9 functions as a molecular hub, recruiting the replication machinery via interactions with ICP8 (19, 21, 23, 24), UL42 (25–27), and UL8 (28), while engaging cellular proteins (12) such as topoisomerase I (24), hTid-1 (29), and heat shock proteins (30) to regulate its own functions. These multifaceted roles establish UL9 as the central coordinator of HSV-1 DNA replication initiation.

The execution of these initiator functions relies heavily on the specific targeting of replication origins, whose architectures vary significantly across the *Herpesviridae* family. In HSV-1, *oriS* possesses a typical symmetric arrangement, with directional Box I and Box II located on both sides of a central A + T rich spacer, specifically recruiting the viral OBP UL9 to drive lytic replication. This differs from the well-characterized replication origin *oriP* of γ-herpesviruses, which features a bipartite, asymmetric structure containing the family of repeats (FR) and dyad symmetry (DS) elements (31, 32). Studies have shown that *oriP* coordinates replication primarily by using the non-helicase protein EBNA1 as a molecular scaffold to hijack the host ORC and MCM2–7 complexes (33). This mechanism is completely distinct from that of HSV-1 *oriS*, which utilizes a symmetric layout and requires UL9 to form a functional homodimer for target binding and replication initiation.

**Fig 1:**
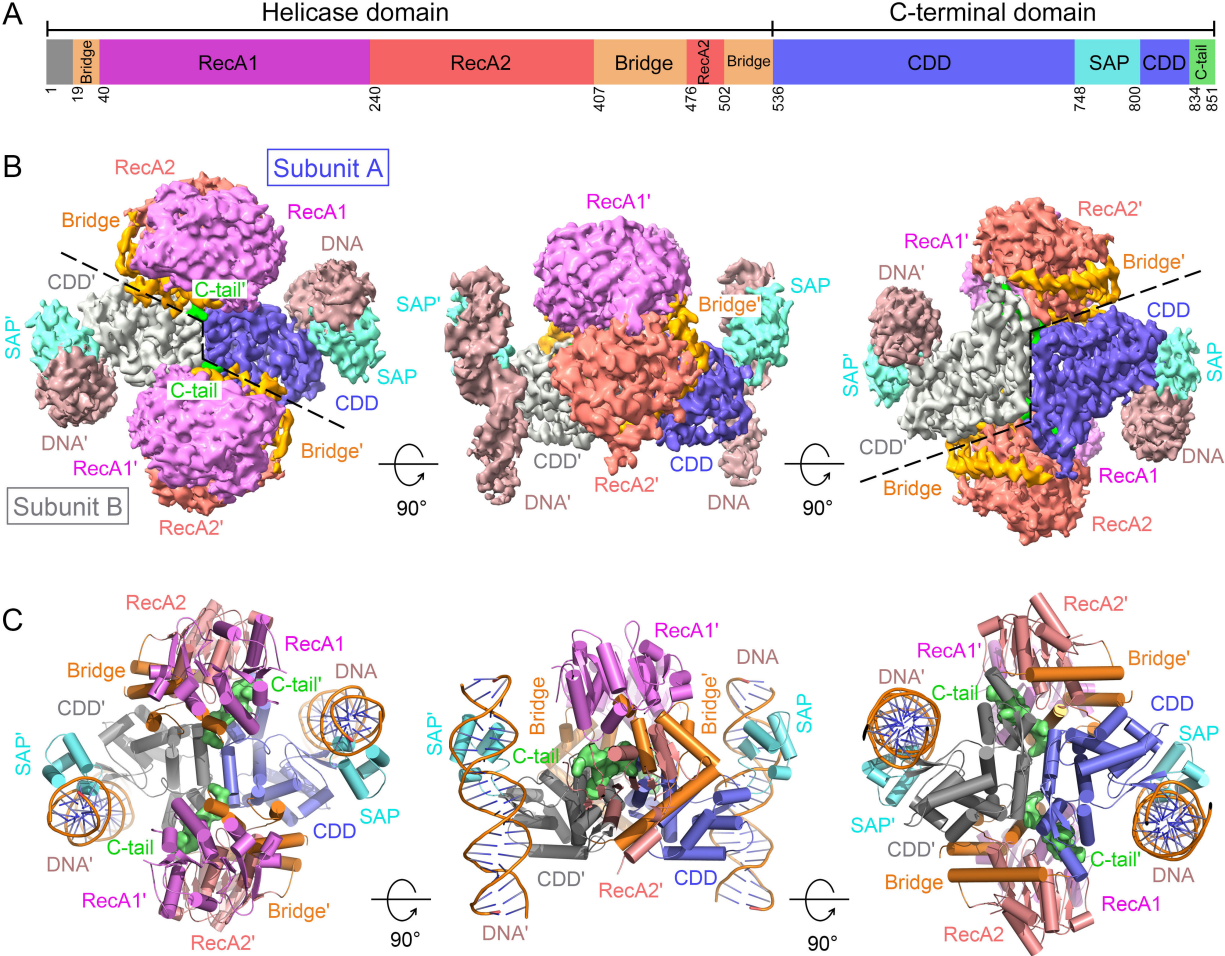
Cryo-EM structure of the UL9–DNA complex. (**A**) Domain architecture of HSV-1 UL9 protein, with the N-terminal helicase domain and C-terminal domain indicated. The RecA1, RecA2, and Bridge subdomains within the helicase domain, alongside the C-terminal dimerization domain (CDD), the SAF-A/B, Acinus and PIAS (SAP) subdomain, and the C-terminal tail (C-tail) within the C-terminal domain, are colored in violet, salmon, orange, slate, aquamarine, and lime, respectively. Domain boundaries are marked with residue numbers. (**B**) Three orthogonal views of the cryo-EM density map for the UL9–DNA complex, colored according to the domain coloring scheme in panel A. (**C**) Corresponding orthogonal views of the atomic model presented in cartoon representation, colored identically to panel B. The bound DNA duplexes (23-bp dsDNA segments modeled into the density) are shown in brown.

Physiologically, HSV-1 UL9 functions as a homodimer (17, 34), with each protomer comprising an N-terminal catalytic helicase domain and a C-terminal origin-binding domain (Fig. 1A). As a prototypical member of the superfamily 2 (SF2) helicase family, the catalytic core of the UL9 helicase domain is characterized by two tandem, conserved RecA-like subdomains that harbor the signature helicase motifs (Motifs I–VI). Among these, the Walker A (Motif I) and Walker B (Motif II) clusters form the functional asymmetric pocket required for ATP binding and hydrolysis, a process tightly coupled to nucleic acid translocation and duplex unwinding. Despite its essential role, the molecular mechanisms of UL9 have remained elusive due to the lack of structural information. Here, we report the structures of HSV-1 UL9 in both apo and DNA-bound states, determined by cryo-electron microscopy (cryo-EM) single-particle analysis. Combined with *in vitro* biochemical and enzymatic assays, we elucidate the molecular basis of UL9’s dimerization, origin recognition, and ICP8-mediated activity regulation. These findings not only advance our understanding of the mechanism of HSV-1 replication initiation but also provide potential new targets for the development of antiherpesviral drugs.

## RESULTS

### Cryo-EM structure determination of HSV-1 UL9

To determine the structure of HSV-1 UL9, the full-length protein (UL9^FL^) was expressed in insect cells and purified to homogeneity using a tandem two-step purification strategy (Fig. S1A through C). The UL9–DNA complex was prepared by incubating affinity-purified UL9 with 56-bp dsDNA containing *oriS* Box I and II at a molar ratio of 1:1.5, followed by gel filtration (Fig. S1A and D). Using cryo-EM single-particle analysis, the structures of apo-UL9 and the UL9–DNA complex were resolved at global resolutions of 4.1 Å and 3.7 Å, respectively (Fig. 1; Fig. S2 and S3; Table S1). During extensive 2D classification and 3D heterogeneous refinement of both datasets, the particles converged exclusively into dimeric architectures; no monomeric or alternative oligomeric species were observed. Due to the higher resolution of the map, the model of the UL9–DNA complex was first built with the aid of AlphaFold prediction (35); this model was primarily used for subsequent structural analyses. The final model of the UL9–DNA complex comprises residues 19–843 and two DNA duplexes although flexible loops (residues 135–146, 267–283, and 431–456) remain unresolved (Fig. 1C; Fig. S4A). The DNA-binding region (residues 748–799) and the bound DNA exhibited significant flexibility, limiting the local resolution to approximately 4.5 Å (Fig. S2G). Consequently, we modeled this domain and a 23-bp dsDNA segment by rigid-body fitting of the AlphaFold-predicted structure into the density map. While the specific nucleotide sequence in the model remained undetermined, preventing analysis of sequence-specific recognition mechanisms, the overall architecture is well defined. In contrast to the DNA-bound state, residues 748–799 in the apo-UL9 structure were disordered and could not be modeled due to a lack of defined density (Fig. S4B). As illustrated in the schematic and cartoon representations of Fig. 1A, each UL9 protomer is modularly organized into an N-terminal helicase domain—comprising the RecA1, RecA2, and Bridge subdomains—and a C-terminal domain composed of the C-terminal dimerization domain (CDD), the SAF-A/B, Acinus, and PIAS (SAP) subdomain, and the C-terminal tail (C-tail). The structural features and functional implications of these individual subdomains are described in detail in the following sections.

### Architecture of the UL9 dimeric complex

**Fig 2:**
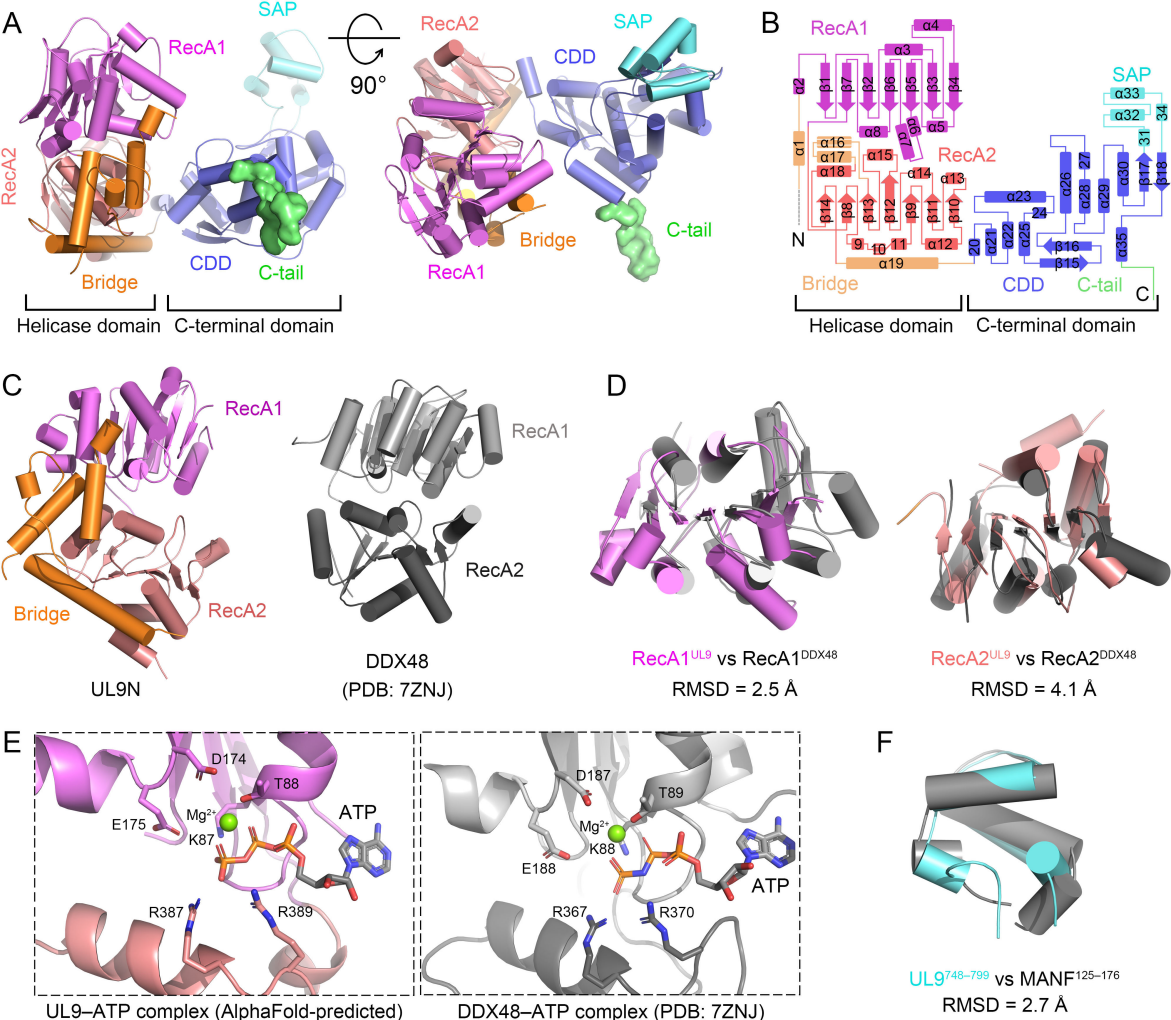
Structural analysis of UL9 protomer. (**A**) Two orthogonal views of the UL9 protomer structure. (**B**) Two-dimensional topology diagram of UL9. (**C**) Structural comparison of UL9 helicase domain with its structural‌ counterpart‌ DDX48 identified by the Dali server. (**D**) Structural superposition of UL9 RecA1 and RecA2 subdomains with corresponding subdomains in DDX48. (**E**) Key residues involved in ATP binding and hydrolysis are‌ conserved‌ between UL9‌ and‌ its structural‌ counterpart‌ ‌DDX48‌.‌ The UL9–ATP complex model was predicted with AlphaFold. (**F**) Structural superposition of the UL9 SAP subdomain with its counterpart MANF identified by the Dali server. All structural representations throughout the panels follow the color scheme in Fig. 1A, with domains labeled and structurally similar proteins (DDX48 and MANF) shown in gray. RMSD values for structural superpositions in panels D and F are provided.

Consistent with previous studies demonstrating that UL9 functions as a dimer in solution and *in vivo* (17, 34), both our apo and DNA-bound structures reveal a *C*_2_-symmetric dimer (Fig. S2; Fig. S4A through C). This dimeric state represents the exclusive functional assembly observed across all our experimental conditions. The single elution peak in size-exclusion chromatography (SEC) profiles echoes the uniform dimeric structures resolved by our cryo-EM analysis, with no detectable monomers or other intermediates under our assayed conditions. The UL9–DNA complex measures approximately 115 × 110 × 65 Å (Fig. S4A). In the apo state, the DNA-binding elements are disordered, resulting in a more slender yet compact structural core (Fig. S4B). Despite the compact dimeric core, individual protomers adopt an extended architecture (Fig. 2A and B; Fig. S4D) that clearly delineates the N-terminal helicase domain (residues 19–535) and C-terminal domain (CTD, residues 536–851). Dimerization is mediated primarily by closely apposed CTDs, which form a rigid central core flanked by two helicase domains; notably, the helicase domains do not interact with one another. Apart from the ordering of the DNA-binding elements, DNA binding induces no major global conformational changes (Fig. S4C).

### Structural details of UL9 helicase domain

Structural similarity analysis of the N-terminal helicase domain using the Dali server (36) identified several cellular DExD/H-box SF2 helicase superfamily members as the top structural matches, including DDX48 (37), DDX17 (38), and Vasa (39) (Table S2; Fig. 2C). Consistent with other SF2 members, UL9’s helicase domain can be subdivided into two RecA-like subdomains: RecA1 (residues 40–242) and RecA2 (residues 243–406 and 476–501) (Fig. 1A; Fig. 2A through D). Superposition of these subdomains with their counterparts in DDX48 (RecA1, residues 23–242; RecA2, residues 243–404) revealed root-mean-square deviation (RMSD) values of 2.5 Å and 4.1 Å (Fig. 2D), respectively, confirming that UL9 retains the conserved RecA-like architecture characterized by seven parallel β-strands. Furthermore, structural alignment of an AlphaFold-predicted UL9–ATP model with DDX48 indicates that key residues involved in ATP binding and hydrolysis within RecA1 are identical (Fig. 2E). However, the UL9 helicase domain also contains additional structural elements (residues 19–39, 407–475, and 502–535) outside the conserved RecA1 and RecA2 subdomains (Fig. 2D). The discontinuous segments assemble into a distinct structural feature spanning RecA1 and RecA2, which we term the Bridge motif (Fig. 2A through C). As detailed below, the Bridge motif participates in UL9 dimerization and likely contributes to allosteric regulation of UL9 function. This unique structural feature distinguishes UL9 from canonical SF2 helicases, particularly the DExD/H-box subfamily, suggesting that herpesvirus UL9-like helicases have evolved specific structural adaptations to support novel viral functions, potentially through novel molecular mechanisms distinct from those of cellular SF2 helicases.

### Structural details of UL9 C-terminal domain

The CTD is structurally organized into three subdomains (Fig. 1, 2A and B). The first (residues 536–747 and 800–833), comprising 12 α-helices and two antiparallel β-sheet pairs, constitutes the structural core of the CTD and mediates UL9 dimerization through extensive interactions with the opposing protomer. Based on its function, we designate it the C-terminal dimerization domain (CDD). The second subdomain (residues 748–799) primarily comprises four α-helices. Structural similarity analysis using the Dali server revealed that this is the only region of the CTD with significant similarity to known structures, with the top structural counterparts all classified as SAP (SAF-A/B, Acinus, PIAS) domains, including MANF (40), CDNF (41), PIAS1 (42), and so on (Table S2; Fig. 2F; Fig. S5A). Notably, these matches exhibited <15% sequence identity despite structural conservation; for instance, the identity between UL9 residues 748–799 and the MANF SAP domain‌ is only 12.8%. The SAP domain, named after three founding members SAF-A/B, Acinus and PIAS, represents a compact 35-residue module characterized by a conserved helix-turn-helix fold (43). It is widely found in proteins involved in chromatin organization, transcriptional regulation, DNA repair, and RNA metabolism and is well-characterized for its DNA/RNA-binding properties (43, 44) (Fig. S5B). Given that this subdomain in UL9 functions as the determinant for dsDNA binding, we classify it as a SAP subdomain (43). The third region comprises the final 17 residues (residues 834–851) located at the extreme C-terminus, which protrudes from the core CDD. Due to its flexible, tail-like conformation, we term this extension the C-terminal tail (C-tail). As detailed below, the C-tail facilitates UL9 dimerization, mediates ICP8 interactions, and modulates UL9 activity.

### Structural details of UL9 dimerization

Under physiological conditions, UL9 functions as a dimer (17, 34). However, the specific dimerization mechanism has remained unclear due to limited structural information (34). In this study, purified UL9 exists definitively as a dimer in solution, in both apo and DNA-bound states, as confirmed by cryo-EM (Fig. 1B and C; Fig. S2). Moreover, *oriS* DNA binding induces no observable global conformational changes in the UL9 dimer structure (Fig. S4C). Structural analysis reveals‌ that UL9 homodimerization is mediated primarily by symmetric interactions between the CDD and C-tail of one protomer and the CDD and helicase domain of the opposing protomer, creating an overall interface area of approximately 3,815 Å^2^. Notably, no direct interprotomer interactions are observed between the helicase domains, and the SAP subdomain is not involved in dimer formation.

**Fig 3:**
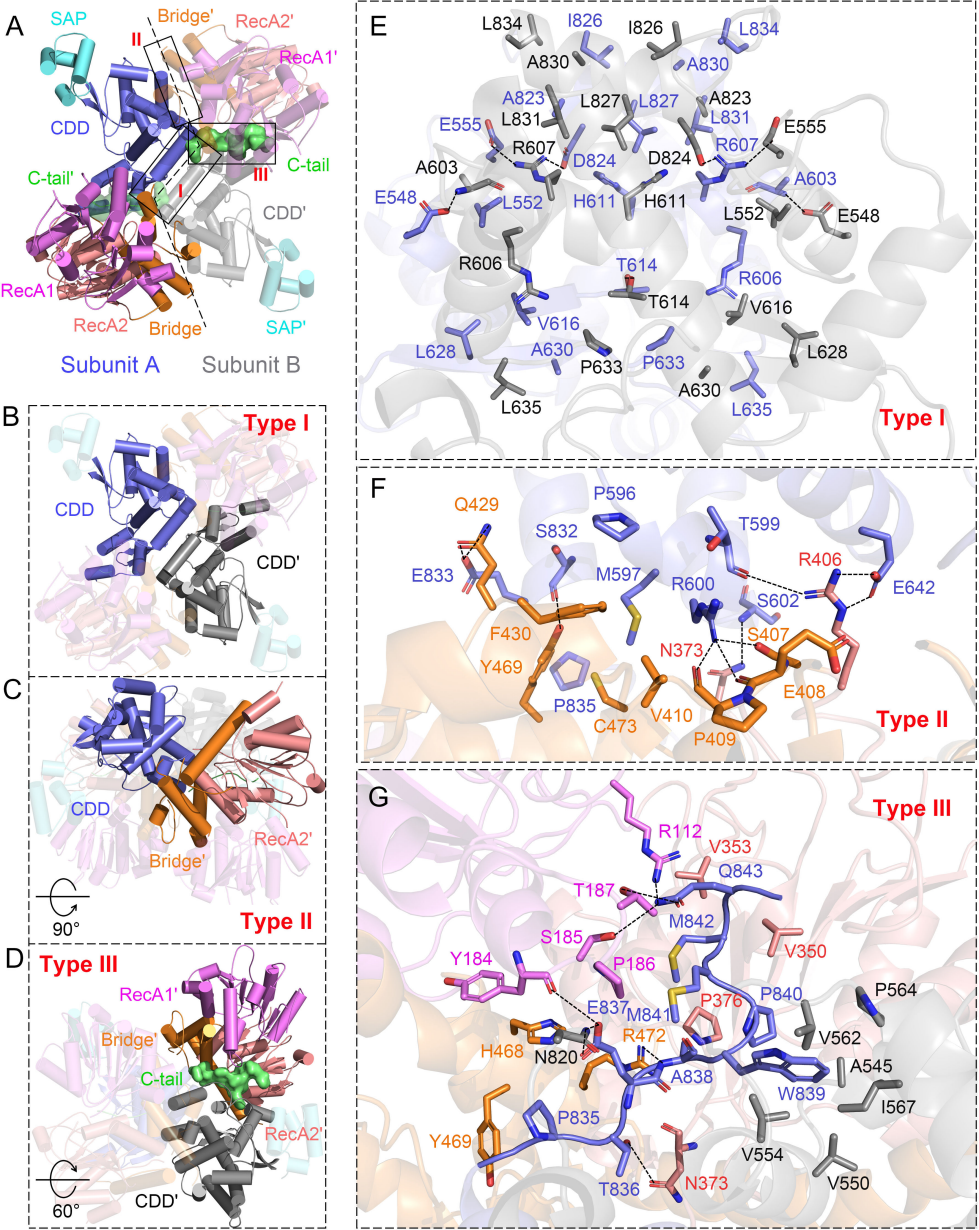
Structural details of UL9 dimerization interfaces. (**A**) Overview of the three types of dimerization interfaces‌ ‌classified‌ in the HSV-1‌ UL9‌ homodimer. The two protomers (Subunit A and Subunit B) are separated‌ by‌ ‌a‌ black‌ dashed line, with individual interfaces highlighted‌ ‌by‌ solid rectangles and labeled (I, II, and III). (**B through D**) Global views of the three interaction interfaces: the symmetrical Type I interface formed between the CDDs (**B**), the Type II interface involving the CDD of one protomer and the RecA2 subdomain and Bridge motif of the opposing protomer (**C**), and the Type III interface comprising the C-tail of one protomer and multiple subdomains (RecA1, RecA2, Bridge, and CDD) of the opposing protomer (**D**). Subdomains directly involved in the dimer contacts are represented as solid cartoons with labels, whereas non-interacting regions are rendered as semi-transparent cartoons for clarity. Rotation symbols indicate the relative orientations to panel A. (**E through G**) Detailed stick representations showing the specific residue interactions within the Type I (**E**), Type II (**F**), and Type III (**G**) interfaces. In panels E to G, the protein backbone structures are represented as cartoons colored by domains as in Fig. 1A. Side chains of key residues in the interactions are displayed as sticks and labeled. Oxygen, nitrogen, and sulfur atoms are colored red, blue, and yellow, respectively; salt bridges and hydrogen bonds are indicated by black dashed lines.

Based on structural features, we defined and designated the ‌dimerization‌ interface‌ into three distinct types specific to this study (Fig. 3A through D; Table S3). The type I interface constitutes the *C*_2_-symmetric dimer core formed by two adjacent CDDs (Fig. 3B). This interface is stabilized mainly by a network of hydrophobic ‌interactions and specific polar contacts (Fig. 3E). The type II interface is‌ formed between the‌ CDD of one protomer and the helicase domain of the opposing protomer (Fig. 3C). Non-polar residues of the CDD make van der Waals contacts with specific hydrophobic residues within the Bridge motif, while its polar residues form salt bridges or hydrogen bonds with N373 of RecA2 and the unique Bridge motif (Fig. 3F). The type III interface is established by the insertion of C-tail into the interdomain cleft formed by the helicase domain and the CDD of the opposing protomer (Fig. 3D). Structurally, the C-tail serves as a “molecular glue” that simultaneously tightly bridges multiple subdomains (RecA1, RecA2, Bridge, and CDD) of the opposing protomer and potentially restricting intersubdomain flexibility (Fig. 3G). This symmetric interaction likely stabilizes the UL9 dimer conformation and contributes to allosteric regulation of its enzymatic activity.

### Interaction details between UL9 and dsDNA

The HSV-1 genome contains three distinct origin sites for viral DNA replication (10, 45). UL9 specifically recognizes and binds to these sequences to initiate DNA synthesis (15, 45, 46). To elucidate the molecular mechanism of the UL9–origin interaction, we determined the cryo-EM structure of the UL9–DNA complex. Notably, the structure reveals that the UL9 dimer binds to dsDNA in a 2:2 stoichiometric ratio (Fig. 1B and C). Compared with apo-UL9, DNA binding induces no significant global conformational changes, except for the ordering of the SAP subdomain (Fig. S4A through C). The structure clearly reveals that dsDNA binding is mediated primarily by the SAP subdomain of the CTD, in full alignment with previous reports (47, 48) (Fig. 1B and C; Fig. S6A and B). Intriguingly, although the helicase domain is predicted to possess nucleic acid-binding capability (Fig. S6C), no specific DNA interactions with this domain were detected in our structure.

**Fig 4:**
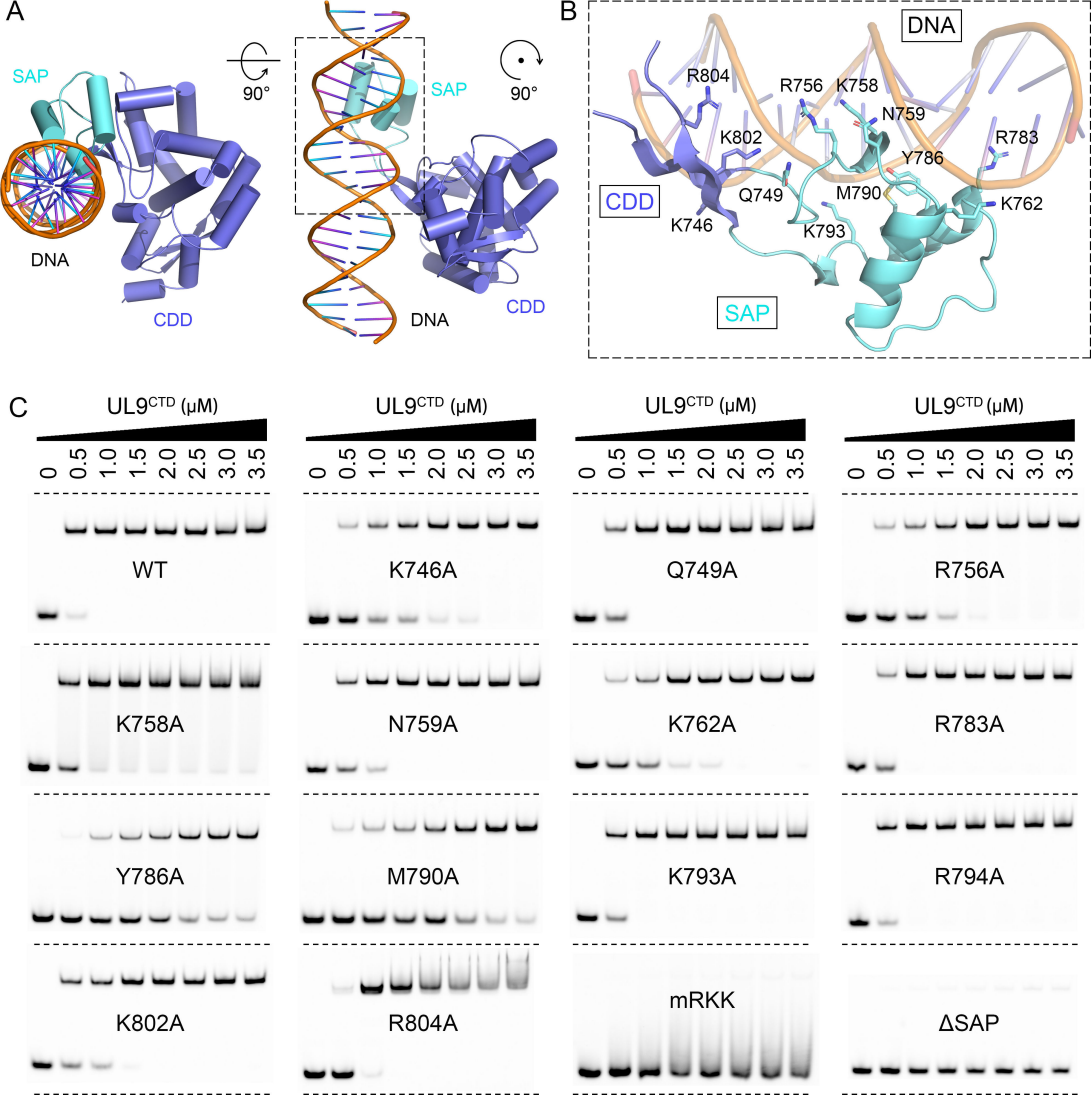
Details of the interaction between UL9 and DNA. (**A**) Two orthogonal views of the core structure of UL9–DNA interaction. (**B**) Structural details of UL9–DNA interaction. Panel B shows a magnified view of the region boxed by a dashed rectangle in panel A after rotation. Protein and DNA structures are represented as cartoons, with side chains of residues involved in UL9–DNA interactions displayed as sticks. (**C**) EMSA analysis of wild-type UL9^CTD^ and its potential DNA-binding mutants. For EMSA assay, a series of seven increasing UL9^CTD^ concentrations (ranging from 0.5 to 3.5 μM) were tested. All assays were independently repeated at least three times with consistent results.

Despite attempts to determine the sequence-specific recognition mechanism of UL9, the flexibility of the SAP–DNA interface limited the local resolution to approximately 4.5 Å, preventing the acquisition of interaction details at the atomic level (Fig. S2G). Rigid-body fitting of the AlphaFold-predicted model into the density map suggests that UL9 likely interacts with dsDNA through residues Q749, R756, K758, N759, K762, R783, Y786, M790, and K793 of the SAP subdomain and residues K746, K802, and R804 of the CDD (Fig. 4A; Fig. S6A). Predicted interactions include ionic contacts with the phosphodiester backbone mediated by basic residues (Arg/Lys) (R756, K762, R783, and K793 of the SAP subdomain and K746, K802, and R804 of the CDD), hydrogen bonds with nucleobases formed by Q749, N759, and K758, and van der Waals stacking interactions involving Y786 and M790 (Fig. 4B).

**Fig 5:**
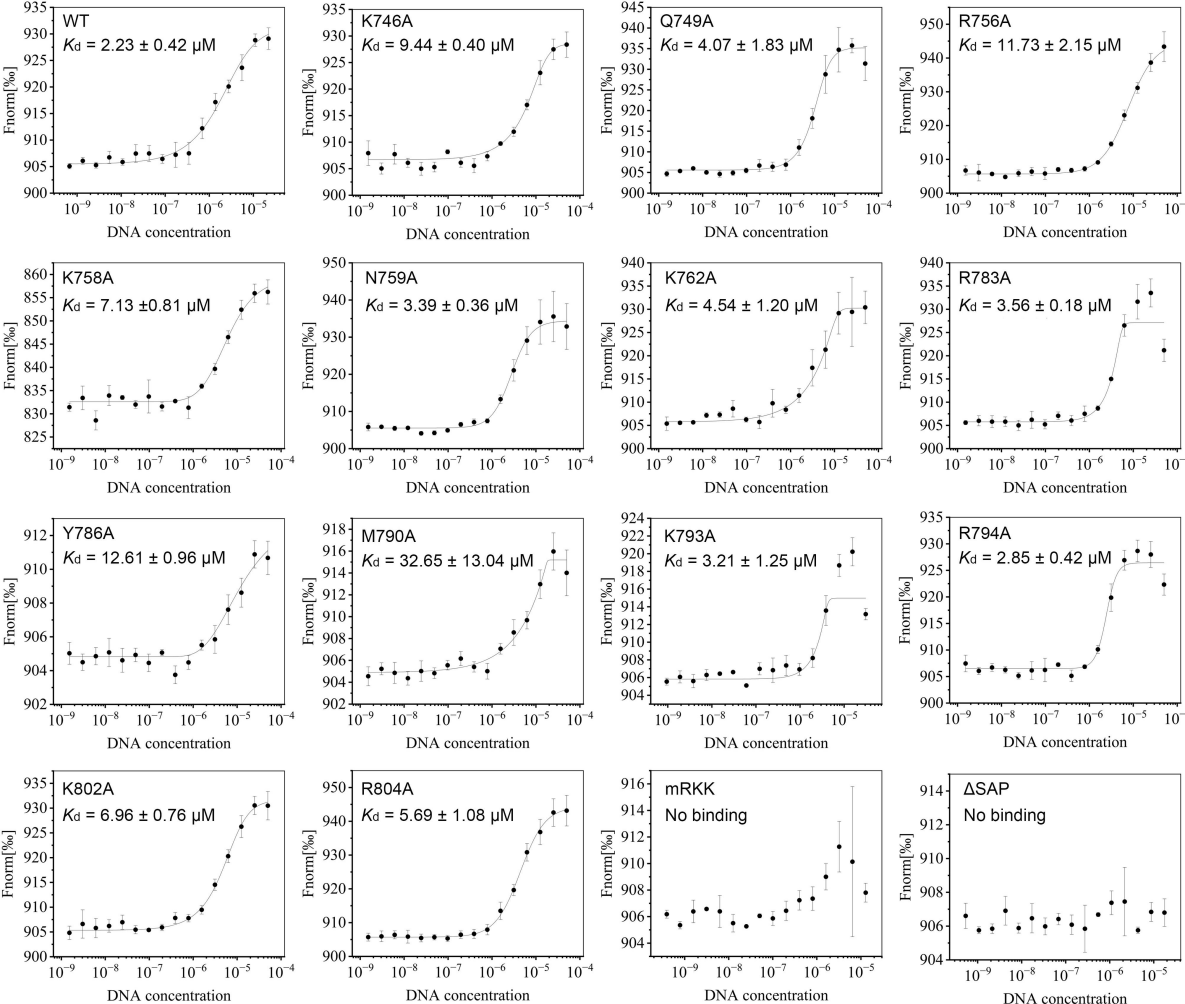
Microscale thermophoresis (MST) analysis of DNA binding by UL9^CTD^ and its mutants. ‌The mutants and their corresponding dissociation constants (*K*_d_) values are indicated in the upper-left corner of each MST assay profile. “No binding” indicates no interaction was detected between UL9^CTD^ and DNA. All assays were repeated independently at least three times with consistent results.

To validate these interactions, we employed electrophoretic mobility shift assays (EMSA) for the visual confirmation and characterization of protein-DNA complex formation and microscale thermophoresis (MST) assays to obtain quantitative affinity measurements (dissociation constants, *K*_d_) based on changes in thermophoretic properties. Given that the origin DNA binding is mediated primarily by the SAP subdomain within the CTD, all assays were performed using the C-terminal domain constructs of UL9 (UL9^CTD^ or UL9C) and its derived mutants. Of note, size-exclusion chromatography (SEC) analysis confirmed that the purified UL9^CTD^ fragment exists predominantly as a monomer in solution, fully consistent with earlier literature (47). The dsDNA substrate used in EMSA and MST assays consisted of a 15-bp Box I sequence from *oriS* (Fig. S1D). The results of EMSA (Fig. 4C) demonstrated that the deletion of the SAP subdomain (UL9C^ΔSAP^) or the triple mutant R756A/K758A/K762A (UL9C^mRKK^) completely abolished the UL9^CTD^–DNA interaction. Single-point mutants K746A, R756A, K758A, K762A, Y786A, and M790A exhibited significant binding attenuation, whereas the Q749A, N759A, R783A, K793A, K802A, and R804A mutants showed moderate but significant reductions. Quantitative MST assays (Fig. 5) confirmed that wild-type UL9^CTD^ binds to dsDNA with a dissociation constant (*K*_d_) of 2.23 ± 0.42 µM. Consistent with EMSA, no binding was detected for either UL9C^ΔSAP^ or UL9C^mRKK^ (Fig. 5). Affinities for mutants R756A, Y786A, and M790A decreased by approximately 5- to 14-fold; mutants K746A, K758A, K762A, K802A, and R804A showed 2- to 4-fold reductions; and mutants Q749A, N759A, R783A, and K793A exhibited 1.4- to 1.8-fold reductions. The binding ability of mutant R794A was almost unaffected due to its distal location within the DNA-binding interface. The results of the MST assays are consistent with those of EMSA, jointly identifying the residues involved in UL9–DNA interactions. Collectively, these data support a coordinated mechanism involving the SAP subdomain and three adjacent CDD residues (K746, K802, and R804). These elements form a stable, triangular SAP–CDD–dsDNA interaction geometry (Fig. 4B) that likely stabilizes the SAP subdomain, facilitating its visualization in the cryo-EM structure.

### Structural basis underlying UL9–ICP8 interaction

**Fig 6:**
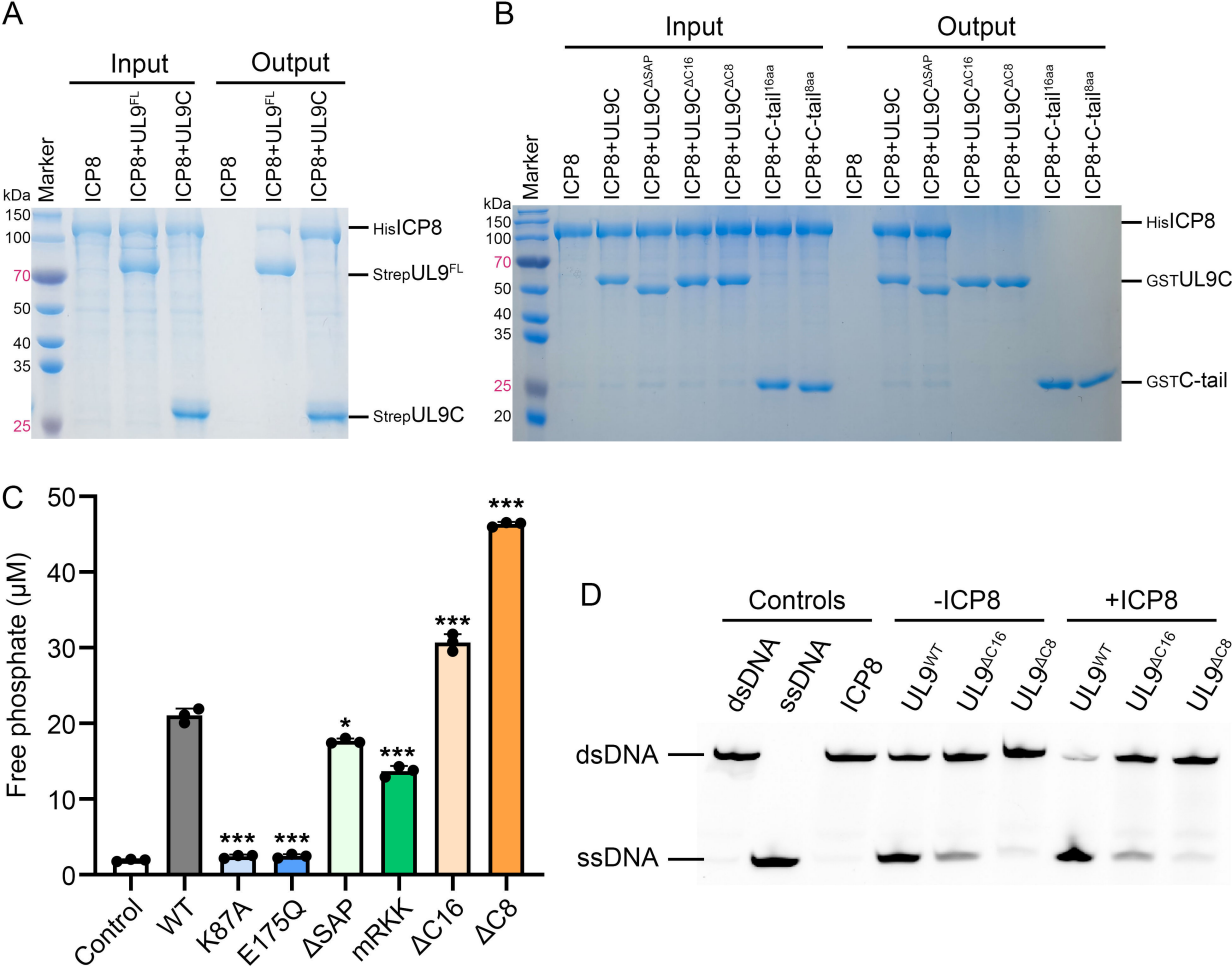
Interaction between UL9 and ICP8. (**A**) Strep-tag pull-down assay demonstrating interaction of His-tagged ICP8 (~128 kDa, prey) with either Strep-tagged full-length UL9 (UL9^FL^, ~95 kDa) or the UL9 C-terminal domain (UL9C, ~32 kDa). (**B**) GST pull-down assay analyzing binding between His-tagged ICP8 and either GST-tagged UL9C variants (around 58 kDa) or the C-tail variants (around 27 kDa); all bait proteins contain a ~26 kDa N-terminal GST tag that accounts for their upward molecular weight shifts. Protein molecular weight markers are indicated on the left of each gel for size verification. (**C**) Comparative ATPase activity of wild-type UL9 vs its functional mutants. ATPase activity assays were performed using the Malachite Green Phosphate Detection Kit. Data are presented as the mean ± s.d. from three independent experiments. Statistical significance was determined by a two-tailed Student’s *t*-test (*P < 0.05; **P < 0.01; ***P < 0.001). (**D**) ICP8-mediated stimulation of UL9 helicase activity. Wild-type UL9 and two C-terminal truncation mutants (ΔC16 and ΔC8) were tested.

UL9 interacts with the ssDNA-binding protein ICP8 to form a ternary replication initiation complex at the origin site (19, 49–51), and ICP8 stimulates the helicase activity of UL9 (19, 21, 23, 50). While previous studies have identified the C-terminus of UL9 as critical in ICP8 binding (19), the precise interaction site and mechanism remain unclear. To map the minimal ICP8-interaction region, we performed *in vitro* pull-down assays using recombinant UL9 truncation mutants guided by the structural information. First, a Strep-tag pull-down assay was employed to compare the binding capacity of UL9^FL^ and UL9^CTD^ for ICP8. The results showed that UL9^FL^ displayed markedly weak interaction with ICP8, whereas UL9^CTD^ exhibited strong binding (Fig. 6A). We next investigated the specific region of UL9^CTD^ participating in this interaction. GST pull-down assays showed that the deletion of either the C-terminal 8 residues (UL9C^Δ8^) or 16 residues (UL9C^Δ16^) completely abrogated the UL9^CTD^–ICP8 interaction, whereas the isolated 8-residues (C-tail^8aa^) or 16-residues (C-tail^16aa^) segments showed no detectable ICP8 binding (Fig. 6B). Furthermore, the deletion of the SAP subdomain did not impair the UL9C–ICP8 interaction, indicating that the SAP subdomain is dispensable for ICP8 binding (Fig. 6B). These results demonstrate that both the CDD and the C-tail are cooperatively required for effective ICP8 binding. This contrast between the weak binding of UL9^FL^ and the strong binding of UL9^CTD^ suggests that the full-length UL9 adopts an auto-inhibited conformation that decreases the interaction with ICP8. Indeed, our structures show that in the full-length UL9 dimer, the C-tail of one protomer is wrapped and partially shielded by the helicase domain of the opposing protomer, thereby occluding the primary ICP8-binding site.

### Allosteric activation of UL9 activity by ICP8 binding

HSV-1 UL9 functions to unwind viral genome DNA at the replication origin through ATP hydrolysis-driven conformational changes, a process facilitated by ICP8 binding (23). Integrating the structural information, we systematically characterized the ATPase and helicase activities of UL9 and specific mutants under various conditions.

Purified recombinant wild-type UL9 exhibits both ATPase and helicase activities (Fig. 6C; Fig. S7A). As expected, the ATP-binding-deficient mutant K87A and hydrolysis-deficient mutant E175Q completely lost both activities, confirming the essential role of ATP hydrolysis (Fig. 6C; Fig. S7A). The DNA-binding-deficient mutants UL9^ΔSAP^ and UL9^mRKK^ retained basal ATPase activity but were completely defective in helicase activity due to loss of DNA binding (Fig. 6C; Fig. S7A). Furthermore, while DNA stimulates the ATPase activity of wild-type UL9, mutants UL9^ΔSAP^ and UL9^mRKK^ fail to respond to DNA stimulation (Fig. S7B). These findings demonstrate that DNA binding is required for helicase activity and stimulates ATP hydrolysis.

We next investigated the impact of the UL9–ICP8 interaction on enzymatic activity. Quantitative ATPase assays showed that the ATPase activity of wild-type UL9 increased in a dose-dependent manner with increasing ICP8 concentrations (Fig. S7C). Compared to wild-type UL9, the C-terminal truncation mutants UL9^Δ8^ and UL9^Δ16^ exhibited constitutively enhanced ATPase activity in the absence of ICP8, with UL9^Δ8^ exhibiting a 2.5-fold increase and UL9^Δ16^ a 1.5-fold increase (Fig. 6C), consistent with previous reports (46). However, increasing ICP8 concentration did not further alter the ATPase activity of either mutant (Fig. S7C). These results indicate that C-terminal truncation increases basal ATPase hydrolysis but abolishes ICP8-dependent regulation. In contrast to the enhancement of ATPase activity, C-terminal truncation progressively impaired helicase activity, with UL9^Δ8^ displaying more severe impairment than UL9^Δ16^ (Fig. S7A). Moreover, while ICP8 markedly stimulated the helicase activity of wild-type UL9, neither truncation mutant responded to ICP8 stimulation (Fig. 6D).

Collectively, these results underscore the critical role of the C-tail in UL9 dimerization, ICP8 interactions, and the allosteric regulation of both ATPase and helicase activities. These findings support‌ the proposed role of the C-tail as a “molecular glue” that bridges multiple subdomains of UL9, limiting intersubdomain movement essential for coupling ATP hydrolysis to DNA unwinding. Because the C-tail interface on the helicase domain overlaps with a potential nucleic acid-binding site, the dynamic interaction between the C-tail and the helicase domain of the opposing protomer governs the modulation of both ATPase and helicase activities.

## DISCUSSION

Our cryo-EM structures of HSV-1 UL9 elucidate distinctive structural features and provide key functional insights into this herpesvirus-specific OBP. Structural similarity analysis confirms that the N-terminal helicase domain of UL9 helicase domain most closely resembles the DExD/H-box SF2 helicases, displaying near-complete conservation of the typical RecA-like fold and key residues involved in ATP binding and hydrolysis. Nevertheless, UL9 exhibits critical deviations from classical superfamily members at both the primary sequence and domain-specific structural levels. On the sequence level, our analysis showing that the canonical Motif II sequence has diverged to “DEVM” in HSV-1 UL9 and “DEIM” in β-herpesvirus homologs, presenting a notable departure from well-characterized SF2 subfamilies such as DEAD-box or DEAH/D-box. On the structural level, the UL9 helicase domain features a unique, evolutionarily derived “Bridge motif” that further distinguishes it from other currently known superfamily members.

The C-terminal domain of UL9 further underscores this structural uniqueness, exhibiting a clear division into three functionally specialized subdomains: the CDD, which primarily mediates dimerization; the SAP subdomain, responsible for sequence-specific recognition of replication origin sites; and the C-tail, which participates in both UL9 dimerization and ICP8 interactions while playing a crucial role in the allosteric regulation of enzymatic activity. While functionally reminiscent simian virus 40 (SV40) large tumor antigen (LTag) (52) and human papillomavirus (HPV) E1 protein (53), which likewise couple origin binding with helicase activities, UL9 functions under a fundamentally different architectural strategy. Unlike these SF3 helicases that assemble into typical functional hexamers (52, 53) via their helicase domains, UL9 functions as a constitutive, strict homodimer mediated by its unique CDD rather than the helicase domain.

Taken together, these distinct structural and mechanistic properties—specifically the atypical “DEVM/DEIM” Motif II and the unique Bridge motif within the helicase domain, the novel CDD-mediated homodimerization architecture rather than classical SF3-like hexamerization, and the specialized origin-recognition SAP domain—clearly distinguish these herpesvirus OBPs from all currently characterized SF2 and SF3 members. Consequently, we propose that UL9-like proteins be formally classified as a separate, independent “UL9-like” subfamily within the SF2 helicase superfamily.

As a distinct “UL9-like” subfamily, these herpesvirus OBPs exhibit a high degree of mechanistic conservation across the *Herpesviridae* family, particularly concerning their homodimerization strategies and origin DNA-binding modalities. In terms of the dimeric architecture, sequence alignment (Fig. S8 and S9) indicates that interfaces I and II are broadly conserved or maintain equivalent biochemical properties across most analyzed α-herpesviruses. In contrast, interface III shows significantly higher conservation, with 11 identical and 7 similar residues. Among human-infecting herpesviruses, a distinct evolutionary partitioning emerges: while interfaces I and II exhibit high conservation within α- or β-herpesviruses individually, a clear divergence is observed between the two lineages. Conversely, interface III retains greater cross-subfamily conservation, preserving 7 identical and 5 similar residues. A critical structural difference is the complete absence of the functionally essential C-tail in β-herpesviruses. Taken together, these observations indicate that while the overarching dimerization framework remains evolutionarily conserved between alpha- and β-herpesviruses, substantial divergence has occurred in mechanistic details, particularly regarding C-tail-mediated dimerization and functional regulation.

This subfamily-specific evolutionary pattern is equally reflected in the origin DNA-binding modules. Sequence alignment reveals remarkable conservation of DNA-binding residues within the SAP subdomains across α-herpesviruses (Fig. S8). Notably, five residues K758, N759, K762, Y786, and M790 are strictly conserved across all species examined, while four residues K746, R756, R783, and K793 consistently maintain positive charges (Arg or Lys). Other residues, such as Q749, K802, and R804, show limited variability, with functionally distinct substitutions observed in 1 or 2 of the 10 analyzed species. When expanding the analysis to human herpesvirus-encoded UL9-like proteins, distinct subfamily-specific conservation patterns are further revealed (Fig. S9). While residues K762 and K802 are identical, and residues K758, R783, and K793 invariably carry positive charges across both subfamilies, N759 shows high conservation in five out of six species, whereas other functiona residues display‌ clear divergence between α- and β-herpesvirus lineages. These findings suggest that while the core electrostatic and spatial requirements for origin recognition are structurally preserved, the SAP subdomains have underwent fine-tuned evolutionary adaptations to accommodate the divergent genomic architectures of their respective viral subfamilies.

As an SF2 member, the UL9 helicase domain possesses potential nucleic acid-binding capability (Fig. S6C). While our structure reveals that primary dsDNA binding is mediated by the SAP subdomain, this configuration alone cannot fully explain the duplex DNA unwinding mechanism. We, therefore, propose that the helicase domain harbors a secondary DNA-binding site with specificity for ssDNA, as suggested by previous studies (54, 55). This putative site likely cooperates with SAP subdomain to facilitate DNA unwinding via ATP hydrolysis-induced conformational changes.

During the preparation of this manuscript, Gustavsson et al. (56) reported cryo-EM structures of UL9–DNA complexes, including dimeric assemblies in the presence and absence of ATPγS, as well as low-resolution models of monomeric and tetrameric (dimer-of-dimer) forms. Their findings regarding UL9 dimerization, sequence-specific DNA binding, and the regulatory role of the C-terminal tail in modulating interactions with ICP8 and UL9 helicase activity align closely with our observations. However, whereas Gustavsson et al. proposed an asymmetric 2:1 (dimer:DNA) stoichiometry for the principal dimeric complex, our results robustly define a symmetric 2:2 complex (Fig. S10). We attribute this difference arises primarily from variations in nucleic acid saturation during sample preparation. Our preparations employed a 1.5-fold molar excess of DNA over UL9 monomer to ensure stoichiometric saturation, whereas the prior study used a 1.2-fold excess. Given that the UL9 homodimer comprises two equivalent protomers, the simultaneous binding of two DNA duplexes is biochemically plausible *in vitro*. Re-inspection of the published density maps (EMD-52135, PDB: 9HGI; EMD-52145, PDB: 9HGJ) from Gustavsson et al. (56) at lower contour levels suggests the presence of residual, unmodeled density for a second DNA duplex (Fig. S10A). Furthermore, their reported tetrameric (dimer-of-dimer) assembly (EMD-52147) appears compatible with a 2:2 binding mode per constituent dimer (Fig. S10A). Mechanistically, a 2:2 stoichiometry enables a single dimer to bridge two distinct boxes within the same origin, providing a model whereby a single dimer can cooperatively coordinate multiple origin elements to drive DNA unwinding.

Beyond these structural comparisons, our work explicitly classifies the DNA-binding region as a SAP subdomain—a conserved eukaryotic fold characteristic of proteins involved in DNA and RNA metabolism. We validated the functional necessity of this domain through comprehensive EMSA and MST mutagenesis of critical residues. Crucially, our apo-state structure reveals that the SAP subdomain is intrinsically flexible, adopting an ordered conformation only upon DNA binding. This conformational plasticity likely promotes efficient sampling and capture of replication origins in solution, followed by stabilization through cooperative interactions with the CDD. Additionally, we investigated the potential function of the helicase domain as a secondary DNA-binding site that collaborates with the SAP subdomain to facilitate origin melting. In parallel, using a series of truncations, we probed possible allosteric modulation of UL9 activity by UL9–ICP8 interactions. In summary, while both studies (56) converge on the fundamental mechanisms of UL9 dimerization and DNA binding, the structural differences most likely represent distinct conformational snapshots captured under varying experimental conditions, thus offering complementary perspectives. Together, these complementary data sets provide a robust foundation for dissecting the molecular mechanisms underlying UL9-mediated replication initiation, which remains incompletely resolved and warrants further investigation.

**Fig 7:**
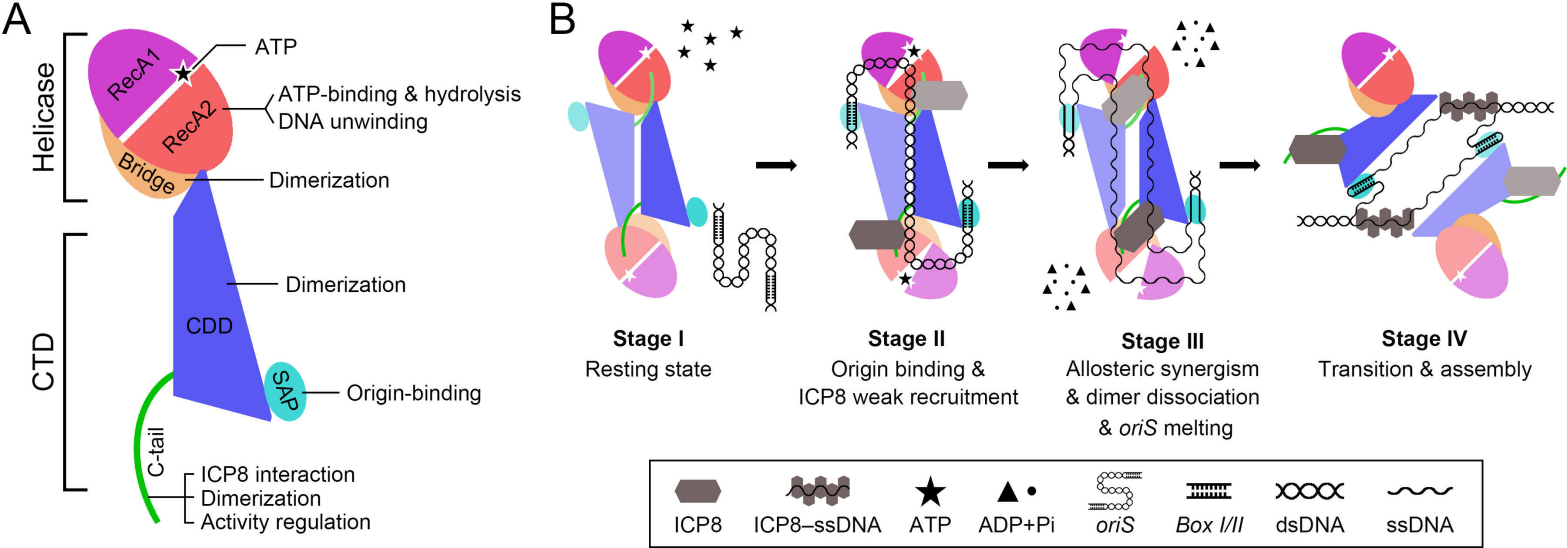
Working model of UL9 function. (**A**) A schematic diagram of HSV-1 UL9 showing domain organization and functional annotation. (**B**) Proposed catalytic cycle of UL9, divided into four stages. Stage 1 (resting state), the apo-UL9 homodimer in a low-activity, autoinhibited conformation. Stage 2 (origin binding and weak recruitment), sequence-specific engagement with *oriS* and basal ATP hydrolysis of UL9 permit transient C-tail liberation, establishing a molecular window that induces the initial, low-affinity weak binding of ICP8 to the SAP subdomain. Stage 3 (allosteric synergism, dimer dissociation, and *oriS* melting), concurrent ICP8 binding and accelerated ATP hydrolysis drive massive, global conformational rearrangements that thoroughly release the C-tail from its helicase binding site, driving the immediate dissociation of the homodimer into highly active monomers while simultaneously executing the initial melting and directional unwinding of the *oriS* duplex. Stage 4 (transition and assembly), transition of the initiation complex to the elongation phase, where the exposed ssDNA template acts as a recruitment platform for downstream replisome components, while a larger cohort of ICP8 molecules cooperatively coats the ssDNA to protect it from degradation. Molecular component annotations are shown in the solid box below.

Based on these structural and biophysical insights, we propose a synergistic allosteric model for the regulation of the UL9–ICP8 interaction. Our SEC data, historical literature (47), and the recent structural capturing of a DNA-bound UL9 monomer by Gustavsson et al. (56) collectively demonstrate that the isolated UL9^CTD^ fragment exists natively as a monomer. By integrating this monomeric property with the dynamic, reciprocal feedback loop between nucleotide turnover and partner recruitment, we propose a refined, four-stage working model for replication initiation (Fig. 7). Stage I (resting state): UL9 predominates as a homodimer with its C-tails sequentially inserted into the interdomain clefts of the opposing protomers. This restrictive spatial arrangement locks the assembly in a low activity, autoinhibited state characterized by basal ATPase activity and severely restricted ICP8-binding capacity. Stage II (origin binding and weak recruitment): upon sequence-specific engagement with *oriS*, the intrinsic basal ATP hydrolysis of UL9 homodimer permits a transient, localized liberation of the C-tail from its inhibitory cleft. This localized conformational fluctuation establishes a molecular window that induces the initial, low-affinity weak binding of ICP8. Stage III (allosteric synergism and concomitant dimer dissociation and *oriS* melting): rather than a linear cascade, the concurrent ICP8 binding and accelerated ATP hydrolysis operate via a powerful reciprocal feedback loop. This synergistic action drives massive, global conformational rearrangements that thoroughly release the C-tail from its helicase-binding site. Consequently, this allosteric collapse destabilizes the quaternary framework, driving the immediate dissociation of the homodimer into monomers while simultaneously executing the initial melting and directional unwinding of the *oriS* duplex. Stage IV (transition and assembly): following local origin melting, the initiation complex transitions to the elongation phase. The newly exposed ssDNA template acts as a crucial recruitment platform, driving the downstream assembly of other essential replisome components that cooperatively engage the UL9–*oriS* complex to initiate processive replication. Concurrently, a larger cohort of ICP8 molecules cooperatively coats the exposed downstream ssDNA to protect it from nuclease degradation and prevent premature reannealing.

In conclusion, this work establishes a rigorous structural framework for understanding UL9 dimerization, SAP-mediated origin recognition, and ICP8-dependent regulation. Nevertheless, the precise molecular dynamics governing sequence-specific origin recognition and unwinding remain to be fully elucidated. Future structural studies of UL9 in complex with origin DNA at distinct conformational states, as well as the high-resolution assembly of the UL9–ICP8–DNA ternary complex, are essential to fully elucidate these mechanisms. Furthermore, because these unique structural features—such as the specialized dimerization interfaces, the plastic SAP subdomain, and the allosteric C-tail—are highly conserved among herpesvirus OBPs yet distinct from host replication machinery, they provide a highly promising strategy for the development of novel antiherpesviral therapeutics.

## MATERIALS AND METHODS

### DNA substrates and preparation

All DNA substrates used in this study were synthesized by Tsingke Biotechnology and purified via HPLC. Oligonucleotides were dissolved in Annealing Buffer (10 mM Tris-HCl, pH 8.0, 50 mM NaCl, 1 mM EDTA) to a stock concentration of 100 μM. For preparation of unlabeled dsDNA or fork DNA, complementary strands were mixed at a 1:1 molar ratio. For FAM-labeled constructs, the labeled and unlabeled complementary strands were mixed at a 1:1.2 molar ratio. Mixtures were denatured at 95°C for 5 min in a thermal cycler (Bio-Rad), followed by gradually cooling to room temperature for at least 3 h with the thermal cycler lid open. Annealed products were stored at 4°C and used within 1 week. The specific sequences of the DNA substrates used for cryo-EM, biochemical assays, and binding studies are listed below (and schematically illustrated in Fig. S1D):

dsDNA substrate containing Box I and II from *oriS* used in cryo-EM single particle analysis:

F_cryo-EM_: 5′-AAGCGTTCGCACTTCGTCCCAATATATATATATTATTAGGGCGAAGTGCGAGCACT-3′

R_cryo-EM_: 5′-AGTGCTCGCACTTCGCCCTAATAATATATATATATTGGGACGAAGTGCGAACGCTT-3′

dsDNA substrate containing Box I from *oriS* used in helicase and ATPase activity assay:

F_Helicase_: 5′-CGCGAAGCGTTCGCACTTCGTCCTTTTTTTTTTTTTTT-3′

R_Helicase_: 5′-TTTTTTTTTTTTTTTGGACGAAGTGCGAACGCTTCGCG-FAM-3′

15-bp dsDNA of Box I from *oriS* for MST assay and EMSA assay:

F_MST_: 5′-GCGTTCGCACTTCGT-3′

R_MST_: 5′-ACGAAGTGCGAACGC-3′

### Protein expression and purification

The genes encoding full-length HSV-1 UL9 (UL9^FL^, UniProt: P10193) and ICP8 (UniProt: P04296) were codon-optimized and synthesized (GeneralBiol). UL9 was cloned into the pFastBac-1 (Invitrogen) vector with an N-terminal Strep-tag (^Strep^UL9) and expressed in Hi5 insect cells. Cells were harvested 48 h post-infection with P3 baculovirus via centrifugation (1,500 × *g*, 10 min). The pellet was resuspended in Lysis Buffer (50 mM HEPES, pH 7.0, 500 mM NaCl, and 10% [vol/vol] glycerol) supplemented with cOmplete (Roche). After sonication for 15 min and centrifugation using a Beckman JA25.50 rotor at 20,000 × *g* for 40 min at 4°C, the resulting supernatant was incubated with Strep-Tactin XT beads (IBA Lifesciences) for 1 h at 4°C. Following three washes with Lysis Buffer, the target protein was eluted using 2.5 mM D-biotin in Lysis Buffer. The target protein was pooled, supplemented with 1 mM DTT, concentrated (50 kDa molecular mass cut-off [MWCO], AmiconUltra), and further purified using a Superdex 200 Increase 10/300 GL column (GE Healthcare) in Gel Filtration Buffer (20 mM HEPES, 300 mM NaCl, 1 mM DTT, pH 7.0).

The C-terminal domain of UL9 (residues 536–851, UL9^CTD^) was cloned into pET-22b vector (Novagen) with an N-terminal 8 × His tag and overexpressed in *E. coli* BL21 (DE3) (Novagen) cells. Protein expression was induced at 16°C for 16–20 h with 0.3 mM isopropyl-1-thio-β-D-galactopyranoside (IPTG) until OD600 ≈ 0.6. The harvested cells were resuspended in Lysis Buffer and lysed by three passes through a microfluidizer. After high-speed centrifugation at 20,000 × *g* for 40 min, the clarified supernatant was incubated with Ni-NTA agarose (GE Healthcare) for 1 h at 4°C. Beads were washed three times with Lysis Buffer containing 60 mM imidazole, and target protein was eluted in Lysis Buffer containing 300 mM imidazole. The eluted protein was pooled, concentrated (10 kDa MWCO), and further purified using Superdex 75 Increase 10/300 GL column (GE Healthcare) in Gel Filtration Buffer. All UL9^CTD^ mutants were purified using the same protocol as wild-type. UL9^CTD^ and its truncation mutants used for GST pull-down assays, along with two C-tail fragments, were cloned into the pGEX-6P-1 vector (GE Healthcare) to generate ‌GST-tagged fusion proteins. Recombinant protein expression, cell resuspension, and lysis procedures followed the same protocol as for His-tagged UL9^CTD^. After centrifugation, the clarified supernatants were incubated with Glutathione-Sepharose 4B beads (GE Healthcare) at 4°C for 1 h. The resin was then washed with Lysis Buffer, and the GST-tagged target proteins were eluted using GST Elution Buffer (50 mM HEPES, 300 mM NaCl, 50 mM reduced glutathione, 1 mM DTT, pH 7.0). Finally, the eluted proteins were analyzed‌ using SDS-PAGE, concentrated, and buffer-exchanged into Gel Filtration Buffer for‌ subsequent analysis. The Strep-tagged UL9^CTD^ construct was generated similar to the His-tagged version, with the N-terminal 8 × His tag replaced by a Strep-tag. Recombinant protein expression, cell harvesting, resuspension, and lysis procedures followed identical protocols as described for His-tagged UL9^CTD^. For affinity purification, the clarified supernatant was incubated with Strep-Tactin XT beads (IBA Lifesciences) for 1 h at 4°C, washed, and eluted using 2.5 mM D-biotin in Lysis Buffer. The eluted target protein was then pooled, concentrated (10 kDa MWCO), and further purified using a Superdex 75 Increase 10/300 GL column (GE Healthcare) in Gel Filtration Buffer. The final purified protein was used for Strep-tag pull-down assays.

The full-length ICP8 was cloned into pFastBac-1 vector with an N-terminal 8 × His tag and overexpressed in insect cells using the same protocol as UL9^FL^. Cell harvesting, resuspension, and lysis also followed the procedure outlined for UL9^FL^. ICP8 was purified via Ni-NTA affinity chromatography similar to UL9^CTD^ and then used for biochemical studies.

To prepare the UL9–dsDNA complex, purified UL9 and pre-annealed dsDNA were mixed at a 1:1.5 molar ratio in 150 mM NaCl and incubated for 1 h on ice. The mixture was further purified using Superdex 200 Increase 10/300 GL column in buffer containing 20 mM HEPES, 150 mM NaCl, 1 mM DTT, pH 7.0 (Fig. S1). Fractions containing the target complex were pooled and concentrated, and the fresh sample was used immediately for structural studies.

### Cryo-EM grid preparation and data collection

For cryo-EM grid preparation, freshly concentrated UL9 and UL9–dsDNA complex were first diluted to 0.6 mg/mL and 0.8 mg/mL, respectively, using a low-salt buffer consisting of 20 mM HEPES, 50 mM NaCl, pH 7.0. The cryo-EM grids were prepared using an automatic plunge freezer Vitrobot Mark IV (Thermo Fisher Scientific) under 100% humidity at 4°C. Each aliquot of 3 μL diluted UL9 or UL9–dsDNA complex was loaded onto the freshly glow-discharged NiTi grids (R1.2/1.3, 300 mesh, Zhenjiang Lehua). After incubation for 10 s, excess sample was blotted with filter paper for 3 s with a blot force of 3, and then the grid was vitrified by flash plunging into liquid ethane. All data sets were automatically collected using SerialEM (57) through the beam-image shift data collection method (58) on a 300 kV Titan Krios microscope (Thermo Fisher Scientific) equipped with an energy filter and a K2 Summit detector (Gatan). Images were recorded under the super-resolution mode at a nominal magnification of 165,000× and a defocus value ranged from –0.8 to –3.0 μm, resulting in a pixel size of 0.41 Å. Each move stack was exposed for 6.2 s with a total exposure dose of ~60 electrons per Å^2^ over 32 frames.

### Data processing and reconstruction

All cryo-EM movie stacks were binned and subjected to beam-induced motion correction and anisotropic magnification correction using MotionCor2 (59). Contrast transfer function (CTF) parameters were estimated with CTFFIND4.1 (60) using non-dose-weighted images. Subsequent processing steps, including particle picking, 2D classification, 3D classification, and auto-refinement, were performed in RELION (61) using dose-weighted images. First, particles were automatically picked using the Laplacian-of-Gaussian filter and processed with several rounds of reference-free 2D classification. Then, the best five images with clear structural features and diverse orientations were selected as templates for reference-based particle auto-picking. For apo-UL9, a total of 1,090,101 particles were picked from 2,080 micrographs and subjected to reference-free 2D classification. A subset of 249,188 particles with clear structural features were kept for further 3D classification with *C*_2_ symmetry imposed, using the initial model generated by RELION. One among the five classes containing 158,949 particles was selected for final 3D auto-refinement, resulting in a 4.1-Å resolution density map (Fig. S2). For the UL9–DNA complex, 913,885 particles were picked from 2,055 micrographs. After 2D classification, 147,241 particles were selected for further 3D classification with C2 symmetry, using the density map of apo-UL9 as the initial model. 110,702 particles from the best class among five were subjected to 3D auto-refinement and resulted in a 3.7-Å resolution density map (Fig. S2). Local resolutions of the final maps were calculated using ResMap (62). Statistics for data collection and processing are summarized in Table S1.

### Model building and refinement

For model building, the UL9–dsDNA complex was first built in Coot (63) with the help of the predicted model from AlphaFold 2 (35) based on its higher resolution and better-quality density map. The cryo-EM density map of C-terminal domain (residues 536–844) in the UL9–dsDNA complex was of sufficient quality to trace almost all the residues. However, some density was missing within local regions of the N-terminal helicase domain. The complete model was further improved by iterative rounds of real-space refinement using PHENIX (64) and manual modification in Coot until no further improvement could be achieved. The apo-UL9 model was generated by docking the refined model of UL9–dsDNA complex into the corresponding density map in UCSF ChimeraX (65), followed by iterative real-space refinement and manual modification. The final model of UL9–dsDNA complex includes residues 19–843 and two duplex DNA molecules, with only residues 135–146, 267–283, and 431–456 unmodeled due to the lack of clear density. In contrast, residues 748–799 in the apo-UL9 model were not modeled due to the absence of corresponding density in the cryo-EM map. Statistics of the final models were summarized in Table S1. Structural analysis was performed with UCSF ChimeraX and PyMOL (https://pymol.org/).

### ATPase activity assay

ATPase activity assays were performed using the Malachite Green Phosphate Detection Kit (Beyotime) according to the manufacturer’s instructions. Briefly, 1 μM of purified UL9 protein was incubated with 2.5 mM ATP in 20 μL reaction volume (ATPase Buffer: 20 mM HEPES [pH 7.5], 150 mM NaCl, 5 mM MgCl_2_, 1 mM DTT, and 10% [vol/vol] glycerol) in 96-well plates and incubated at 37°C for 30 min. Reactions were terminated by the addition of 180 μL of 200 mM EDTA, followed by the addition of 70 μL of malachite green reagent for colorimetric detection. After a further 30-min incubation at room temperature, the absorbance at 630 nm was measured using an EnVision Multimode Microplate Reader (PerkinElmer). The concentration of released phosphate was determined using a standard curve was prepared according to the kit instructions. All assays were independently repeated at least three times.

### Helicase activity assay

FAM-labeled DNA substrates used for helicase activity assays were prepared by annealing a 3′-FAM-labeled oligonucleotide with an unlabeled complementary strand at a molar ratio of 1:1.2. Helicase reactions (20 μL) were performed by mixing 50 nM of dsDNA or fork DNA substrate with varying concentrations of UL9 in Helicase Buffer (20 mM HEPES, pH 7.5, 150 mM NaCl, 1 mM DTT, 10% glycerol, 5 mM MgCl_2_, 0.1% BSA, and 2.5 mM ATP). After incubation at 37°C for 30 min, the reactions were terminated by adding 1 mg/mL proteinase K and incubating for an additional 20 min to degrade the proteins. The samples were then analyzed using 15% Urea-PAGE gels, and fluorescence signals were captured using a ChemiDoc MP Imaging System (Bio-Rad). All assays were independently repeated at least three times.

### Electrophoretic mobility shift assay

To qualitatively assess the DNA-binding ability of UL9^CTD^, electrophoretic mobility shift assays (EMSA) were performed. Briefly, various concentrations (0.5–3.5 μM) of purified UL9^CTD^ or its mutants were incubated with 40 nM FAM-labeled dsDNA for 1 h at 4°C in 20 μL of EMSA Buffer (50 mM Tris, pH 8.0, 50 mM NaCl, 1 mM DTT). Following incubation, samples were mixed with loading dye and analyzed by 7% non-denaturing PAGE in 1 × TG buffer (25 mM Tris and 192 mM glycine). The gels were imaged using a ChemiDoc MP Imaging System (Bio-Rad) according to the manufacturer instructions. All assays were independently repeated at least three times.

### Microscale thermophoresis assay

To quantitatively determine the equilibrium dissociation constant *K*_d_ for the dsDNA-binding affinity of UL9^CTD^, microscale thermophoresis (MST) assay was performed using a Monolith NT.115 instrument (NanoTemper Technologies). Purified UL9^CTD^ or its mutants (ligands) were serially diluted in MST Buffer (50 mM Tris, pH 8.0, 150 mM NaCl, 1 mM DTT and 0.05% [vol/vol] Tween-20) and mixed with 40 nM FAM-labeled dsDNA (target), consistent with the substrates used in the EMSA. After a 30-min incubation in the dark at room temperature, samples were loaded into premium coated capillaries (NanoTemper). Measurements were performed at 25°C with 20% LED power and medium MST power (laser off/on times of 5 s and 10 s, respectively). Data were collected using MO.Control software (v3.0). The normalized fluorescence (F_norm_) was plotted against protein concentration, and *K*_d_ values were calculated by fitting the data to a single-site binding model using MO.Affinity Analysis 3 (v3.0) (NanoTemper). All assays were independently repeated three times.

### Pull-down assays

For GST pull-down assays, 50 μg of purified GST-tagged UL9^CTD^ mutants or C-tail peptides were mixed with an equal amount of purified ICP8. After a portion (10%) of each mixture was reserved as input controls, the remaining samples were combined with 25 μL of pre-equilibrated GST beads (GE Healthcare) and 1 mL Pull-down Buffer (50 mM Tris, pH 8.0, 150 mM NaCl, 1 mM DTT, and 0.05% [vol/vol] Tween-20), followed by incubation with gentle rotation for 1 h at 4 °C. The beads were then pelleted by centrifugation at 1,000 × *g* and washed three times with 1 mL Pull-down Buffer. Bound proteins were eluted by boiling the beads in SDS-PAGE loading buffer and subsequently analyzed by 12% SDS-PAGE with Coomassie blue staining. For Strep-tag pull-down assays, 50 μg of purified Strep-tagged UL9^CTD^ or full-length UL9 was mixed with an equal amount of ICP8. Subsequent steps were performed as described for the GST pull-down assay. All assays were independently repeated three times.

## Data Availability

The cryo-EM density maps and corresponding atomic coordinates have been deposited in the Protein Data Bank (PDB) and Electron Microscopy Data Bank (EMDB) under the following accession numbers, respectively: apo-UL9 (PDB 9VJI, EMD-65114) and the UL9–DNA complex (PDB 9VJH, EMD-65113). All data that support the findings of this study are available from the corresponding authors upon reasonable request.

## SUPPLEMENTAL MATERIAL

Supplemental material jvi.00271-26-s0001.pdf Fig. S1 to S10; Tables S1 to S3.

ASM does not own the copyrights to Supplemental Material that may be linked to, or accessed through, an article. The authors have granted ASM a non-exclusive, world-wide license to publish the Supplemental Material files. Please contact the corresponding author directly for reuse.

## References

[1] Lu L, Fan M, Li X, Su W, Wang X, Xue Y, Ma L, Yao K, Liu Y, Jia L, Yu D. 2025. Herpesvirus-associated diseases: biomarkers and advancements in clinical research. Virol J 22:177. doi:10.1186/s12985-025-02795-7 40457345 PMC12128367

[2] Grinde B. 2013. Herpesviruses: latency and reactivation – viral strategies and host response. J Oral Microbiol 5:22766. doi:10.3402/jom.v5i0.22766 PMC3809354 24167660

[3] Molero García JM, Moreno Guillén S, Rodríguez-Artalejo FJ, Ruiz-Galiana J, Cantón R, De Lucas Ramos P, García-Botella A, García-Lledó A, Hernández-Sampelayo T, Gómez-Pavón J, González Del Castillo J, Martín-Delgado MC, Martín Sánchez FJ, Martínez-Sellés M, Bouza E. 2023. Status of herpes zoster and herpes zoster vaccines in 2023: a position paper. Rev Esp Quimioter 36:223–235. doi:10.37201/req/004.2023 36752132 PMC10238794

[4] Piret J, Boivin G. 2016. Antiviral resistance in herpes simplex virus and varicella-zoster virus infections: diagnosis and management. Curr Opin Infect Dis 29:654–662. doi:10.1097/QCO.0000000000000288 27306564

[5] Prichard MN. 2009. Function of human cytomegalovirus UL97 kinase in viral infection and its inhibition by maribavir. Rev Med Virol 19:215–229. doi:10.1002/rmv.615 19434630 PMC3777615

[6] Whitley R, Baines J. 2018. Clinical management of herpes simplex virus infections: past, present, and future. F1000Res 7:1726. doi:10.12688/f1000research.16157.1 PMC6213787 30443341

[7] Zinser E, Krawczyk A, Mühl-Zürbes P, Aufderhorst U, Draßner C, Stich L, Zaja M, Strobl S, Steinkasserer A, Heilingloh CS. 2018. A new promising candidate to overcome drug resistant herpes simplex virus infections. Antiviral Res 149:202–210. doi:10.1016/j.antiviral.2017.11.012 29155164

[8] Bazhulina NP, Surovaya AN, Gursky YG, Andronova VL, Moiseeva ED, Nikitin CACM, Golovkin MV, Galegov GА, Grokhovsky SL, Gursky GV. 2014. Complex of the herpes simplex virus type 1 origin binding protein UL9 with DNA as a platform for the design of a new type of antiviral drugs. J Biomol Struct Dyn 32:1456–1473. doi:10.1080/07391102.2013.820110 23879454 PMC4066892

[9] Font M, Sanmartín C, Alonso ML, Gracia L, Losa MJ, Marquiegui B, Merino I, Nadal E, Ruiz I, Monge A, Bengoechea MT, Cabodevilla F, Elena S, Martinez-Irujo JJ, Odriozola L, Peñuelas I, Santiago E, Homa F, Wathen MW. 2000. New antiherpetic 1,3-phenylene derivatives, inhibitors of the interaction of the HSV-1 origin binding protein (OBP) with DNA. Drug Des Discov 16:295–315. 10807035

[10] Muylaert I, Tang KW, Elias P. 2011. Replication and recombination of herpes simplex virus DNA. J Biol Chem 286:15619–15624. doi:10.1074/jbc.R111.233981 21362621 PMC3091170

[11] Weller SK, Coen DM. 2012. Herpes simplex viruses: mechanisms of DNA replication. Cold Spring Harb Perspect Biol 4:a013011. doi:10.1101/cshperspect.a013011 22952399 PMC3428768

[12] Packard JE, Dembowski JA. 2021. HSV-1 DNA replication-coordinated regulation by viral and cellular factors. Viruses 13:2015. doi:10.3390/v13102015 34696446 PMC8539067

[13] Weerasooriya S, DiScipio KA, Darwish AS, Bai P, Weller SK. 2019. Herpes simplex virus 1 ICP8 mutant lacking annealing activity is deficient for viral DNA replication. Proc Natl Acad Sci USA 116:1033–1042. doi:10.1073/pnas.1817642116 30598436 PMC6338833

[14] Elias P, O’Donnell ME, Mocarski ES, Lehman IR. 1986. A DNA binding protein specific for an origin of replication of herpes simplex virus type 1. Proc Natl Acad Sci USA 83:6322–6326. doi:10.1073/pnas.83.17.6322 3018724 PMC386495

[15] Olivo PD, Nelson NJ, Challberg MD. 1988. Herpes simplex virus DNA replication: the UL9 gene encodes an origin-binding protein. Proc Natl Acad Sci USA 85:5414–5418. doi:10.1073/pnas.85.15.5414 2840659 PMC281767

[16] Weir HM, Calder JM, Stow ND. 1989. Binding of the herpes simplex virus type 1 UL9 gene product to an origin of viral DNA replication. Nucleic Acids Res 17:1409–1425. doi:10.1093/nar/17.4.1409 2537958 PMC331812

[17] Fierer DS, Challberg MD. 1992. Purification and characterization of UL9, the herpes simplex virus type 1 origin-binding protein. J Virol 66:3986–3995. doi:10.1128/JVI.66.7.3986-3995.1992 1318393 PMC241201

[18] Boehmer PE, Dodson MS, Lehman IR. 1993. The herpes simplex virus type-1 origin binding protein. DNA helicase activity. J Biol Chem 268:1220–1225. doi:10.1016/S0021-9258(18)54063-1 8380408

[19] Boehmer PE, Lehman IR. 1993. Physical interaction between the herpes simplex virus 1 origin-binding protein and single-stranded DNA-binding protein ICP8. Proc Natl Acad Sci USA 90:8444–8448. doi:10.1073/pnas.90.18.8444 8397405 PMC47373

[20] Stabell EC, Olivo PD. 1993. A truncated herpes simplex virus origin binding protein which contains the carboxyl terminal origin binding domain binds to the origin of replication but does not alter its conformation. Nucleic Acids Res 21:5203–5211. doi:10.1093/nar/21.22.5203 8255778 PMC310638

[21] Boehmer PE. 1998. The herpes simplex virus type-1 single-strand DNA-binding protein, ICP8, increases the processivity of the UL9 protein DNA helicase. J Biol Chem 273:2676–2683. doi:10.1074/jbc.273.5.2676 9446572

[22] Bruckner RC, Crute JJ, Dodson MS, Lehman IR. 1991. The herpes simplex virus 1 origin binding protein: a DNA helicase. J Biol Chem 266:2669–2674. doi:10.1016/S0021-9258(18)52296-1 1846632

[23] Arana ME, Haq B, Tanguy Le Gac N, Boehmer PE. 2001. Modulation of the herpes simplex virus type-1 UL9 DNA helicase by its cognate single-strand DNA-binding protein, ICP8. J Biol Chem 276:6840–6845. doi:10.1074/jbc.M007219200 11112774

[24] Makhov AM, Lee S-K, Lehman IR, Griffith JD. 2003. Origin-specific unwinding of herpes simplex virus 1 DNA by the viral UL9 and ICP8 proteins: visualization of a specific preunwinding complex. Proc Natl Acad Sci USA 100:898–903. doi:10.1073/pnas.0237171100 12552114 PMC298698

[25] Monahan SJ, Grinstead LA, Olivieri W, Parris DS. 1998. Interaction between the herpes simplex virus type 1 origin-binding and DNA polymerase accessory proteins. Virology 241:122–130. doi:10.1006/viro.1997.8953 9454723

[26] Trego KS, Parris DS. 2003. Functional interaction between the herpes simplex virus type 1 polymerase processivity factor and origin-binding proteins: enhancement of UL9 helicase activity. J Virol 77:12646–12659. doi:10.1128/jvi.77.23.12646-12659.2003 14610187 PMC262563

[27] Trego KS, Zhu Y, Parris DS. 2005. The herpes simplex virus type 1 DNA polymerase processivity factor, UL42, does not alter the catalytic activity of the UL9 origin-binding protein but facilitates its loading onto DNA. Nucleic Acids Res 33:536–545. doi:10.1093/nar/gki196 15673714 PMC548344

[28] McLean GW, Abbotts AP, Parry ME, Marsden HS, Stow ND. 1994. The herpes simplex virus type 1 origin-binding protein interacts specifically with the viral UL8 protein. J Gen Virol 75:2699–2706. doi:10.1099/0022-1317-75-10-2699 7931156

[29] Eom CY, Lehman IR. 2002. The human DnaJ protein, hTid-1, enhances binding of a multimer of the herpes simplex virus type 1 UL9 protein to oris, an origin of viral DNA replication. Proc Natl Acad Sci USA 99:1894–1898. doi:10.1073/pnas.042689499 11854491 PMC122290

[30] Tanguy Le Gac N, Boehmer PE. 2002. Activation of the herpes simplex virus type-1 origin-binding protein (UL9) by heat shock proteins. J Biol Chem 277:5660–5666. doi:10.1074/jbc.M108316200 11711536

[31] Gahn TA, Schildkraut CL. 1989. The Epstein-Barr virus origin of plasmid replication, oriP, contains both the initiation and termination sites of DNA replication. Cell 58:527–535. doi:10.1016/0092-8674(89)90433-9 2547525

[32] Mei Y, Messick TE, Dheekollu J, Kim HJ, Molugu S, Muñoz LJC, Moiskeenkova-Bell V, Murakami K, Lieberman PM. 2022. Cryo-EM structure and functional studies of EBNA1 binding to the family of repeats and dyad symmetry elements of Epstein-Barr virus *oriP*. J Virol 96:e0094922. doi:10.1128/jvi.00949-22 36037477 PMC9472633

[33] Dhar SK, Yoshida K, Machida Y, Khaira P, Chaudhuri B, Wohlschlegel JA, Leffak M, Yates J, Dutta A. 2001. Replication from oriP of Epstein-Barr virus requires human ORC and is inhibited by geminin. Cell 106:287–296. doi:10.1016/S0092-8674(01)00458-5 11509178

[34] Chattopadhyay S, Weller SK. 2007. Direct interaction between the N- and C-terminal portions of the herpes simplex virus type 1 origin binding protein UL9 implies the formation of a head-to-tail dimer. J Virol 81:13659–13667. doi:10.1128/JVI.01204-07 17942532 PMC2168873

[35] Jumper J, Evans R, Pritzel A, Green T, Figurnov M, Ronneberger O, Tunyasuvunakool K, Bates R, Žídek A, Potapenko A, et al.. 2021. Highly accurate protein structure prediction with AlphaFold. Nature 596:583–589. doi:10.1038/s41586-021-03819-2 34265844 PMC8371605

[36] Holm L. 2022. Dali server: structural unification of protein families. Nucleic Acids Res 50:W210–W215. doi:10.1093/nar/gkac387 35610055 PMC9252788

[37] Pacheco-Fiallos B, Vorländer MK, Riabov-Bassat D, Fin L, O’Reilly FJ, Ayala FI, Schellhaas U, Rappsilber J, Plaschka C. 2023. mRNA recognition and packaging by the human transcription-export complex. Nature 616:828–835. doi:10.1038/s41586-023-05904-0 37020021 PMC7614608

[38] Ngo TD, Partin AC, Nam Y. 2019. RNA specificity and autoregulation of DDX17, a modulator of MicroRNA biogenesis. Cell Rep 29:4024–4035. doi:10.1016/j.celrep.2019.11.059 31851931 PMC6953907

[39] Xiol J, Spinelli P, Laussmann MA, Homolka D, Yang Z, Cora E, Couté Y, Conn S, Kadlec J, Sachidanandam R, Kaksonen M, Cusack S, Ephrussi A, Pillai RS. 2014. RNA clamping by Vasa assembles a piRNA amplifier complex on transposon transcripts. Cell 157:1698–1711. doi:10.1016/j.cell.2014.05.018 24910301

[40] Yan Y, Rato C, Rohland L, Preissler S, Ron D. 2019. MANF antagonizes nucleotide exchange by the endoplasmic reticulum chaperone BiP. Nat Commun 10:541. doi:10.1038/s41467-019-08450-4 30710085 PMC6358605

[41] Yu LY, Viljakainen T, Beckett L, Nam J, Hyysalo A, Lüningschrör P, Albert K, Koppinen T, Hyvärinen T, Tossavainen H, Hellman M, Airavaara M, Sendtner M, Permi P, Lindholm P, Saarma M, Voutilainen MH. 2025. Non-invasive peripheral delivery of CDNF fragment protects neurons in models of Parkinson’s and ALS. bioRxiv. doi:10.1101/2025.02.09.637291

[42] Okubo S, Hara F, Tsuchida Y, Shimotakahara S, Suzuki S, Hatanaka H, Yokoyama S, Tanaka H, Yasuda H, Shindo H. 2004. NMR structure of the N-terminal domain of SUMO ligase PIAS1 and its interaction with tumor suppressor p53 and A/T-rich DNA oligomers. J Biol Chem 279:31455–31461. doi:10.1074/jbc.M403561200 15133049

[43] Aravind L, Koonin EV. 2000. SAP – a putative DNA-binding motif involved in chromosomal organization. Trends Biochem Sci 25:112–114. doi:10.1016/S0968-0004(99)01537-6 10694879

[44] Walker JR, Corpina RA, Goldberg J. 2001. Structure of the Ku heterodimer bound to DNA and its implications for double-strand break repair. Nature 412:607–614. doi:10.1038/35088000 11493912

[45] Olsson M, Tang KW, Persson C, Wilhelmsson LM, Billeter M, Elias P. 2009. Stepwise evolution of the herpes simplex virus origin binding protein and origin of replication. J Biol Chem 284:16246–16255. doi:10.1074/jbc.M807551200 19351883 PMC2713539

[46] Murata LB, Dodson MS. 1999. The herpes simplex virus type 1 origin-binding protein. sequence-specific activation of adenosine triphosphatase activity by a double-stranded DNA containing box I. J Biol Chem 274:37079–37086. doi:10.1074/jbc.274.52.37079 10601266

[47] Martin DW, Muñoz RM, Oliver D, Subler MA, Deb S. 1994. Analysis of the DNA-binding domain of the HSV-1 origin-binding protein. Virology 198:71–80. doi:10.1006/viro.1994.1009 8259684

[48] Stow ND, Hammarsten O, Arbuckle MI, Elias P. 1993. Inhibition of herpes simplex virus type 1 DNA replication by mutant forms of the origin-binding protein. Virology 196:413–418. doi:10.1006/viro.1993.1496 8396795

[49] Manolaridis I, Mumtsidu E, Konarev P, Makhov AM, Fullerton SW, Sinz A, Kalkhof S, McGeehan JE, Cary PD, Griffith JD, Svergun D, Kneale GG, Tucker PA. 2009. Structural and biophysical characterization of the proteins interacting with the herpes simplex virus 1 origin of replication. J Biol Chem 284:16343–16353. doi:10.1074/jbc.M806134200 19329432 PMC2713556

[50] Lee SS, Lehman IR. 1999. The interaction of herpes simplex type 1 virus origin-binding protein (UL9 protein) with Box I, the high affinity element of the viral origin of DNA replication. J Biol Chem 274:18613–18617. doi:10.1074/jbc.274.26.18613 10373472

[51] Boehmer PE, Craigie MC, Stow ND, Lehman IR. 1994. Association of origin binding protein and single strand DNA-binding protein, ICP8, during herpes simplex virus type 1 DNA replication in vivo. J Biol Chem 269:29329–29334. doi:10.1016/S0021-9258(19)62048-X 7961904

[52] Shahid T, Danazumi AU, Tehseen M, Alhudhali L, Clark AR, Savva CG, Hamdan SM, De Biasio A. 2025. Structural dynamics of DNA unwinding by a replicative helicase. Nature 641:240–249. doi:10.1038/s41586-025-08766-w 40108462 PMC12043514

[53] Javed A, Major B, Stead JA, Sanders CM, Orlova EV. 2021. Unwinding of a DNA replication fork by a hexameric viral helicase. Nat Commun 12:5535. doi:10.1038/s41467-021-25843-6 34545080 PMC8452682

[54] Marintcheva B, Weller SK. 2003. Helicase motif ia is involved in single-strand DNA-binding and helicase activities of the herpes simplex virus type 1 origin-binding protein, UL9. J Virol 77:2477–2488. doi:10.1128/JVI.77.4.2477-2488.2003 12551986 PMC141079

[55] Macao B, Olsson M, Elias P. 2004. Functional properties of the herpes simplex virus type I origin-binding protein are controlled by precise interactions with the activated form of the origin of DNA replication. J Biol Chem 279:29211–29217. doi:10.1074/jbc.M400371200 15133043

[56] Gustavsson E, Grünewald K, Elias P, Hällberg BM. 2025. The herpes simplex origin-binding protein: mechanisms for sequence-specific DNA binding and dimerization revealed by Cryo-EM. Nucleic Acids Res 53:gkaf1029. doi:10.1093/nar/gkaf1029 41125242 PMC12543377

[57] Mastronarde DN. 2005. Automated electron microscope tomography using robust prediction of specimen movements. J Struct Biol 152:36–51. doi:10.1016/j.jsb.2005.07.007 16182563

[58] Wu C, Huang X, Cheng J, Zhu D, Zhang X. 2019. High-quality, high-throughput cryo-electron microscopy data collection via beam tilt and astigmatism-free beam-image shift. J Struct Biol 208:107396. doi:10.1016/j.jsb.2019.09.013 31562921

[59] Zheng SQ, Palovcak E, Armache JP, Verba KA, Cheng Y, Agard DA. 2017. MotionCor2: anisotropic correction of beam-induced motion for improved cryo-electron microscopy. Nat Methods 14:331–332. doi:10.1038/nmeth.4193 28250466 PMC5494038

[60] Rohou A, Grigorieff N. 2015. CTFFIND4: fast and accurate defocus estimation from electron micrographs. J Struct Biol 192:216–221. doi:10.1016/j.jsb.2015.08.008 26278980 PMC6760662

[61] Zivanov J, Nakane T, Forsberg BO, Kimanius D, Hagen WJ, Lindahl E, Scheres SH. 2018. New tools for automated high-resolution cryo-EM structure determination in RELION-3. eLife 7:e42166. doi:10.7554/eLife.42166 30412051 PMC6250425

[62] Kucukelbir A, Sigworth FJ, Tagare HD. 2014. Quantifying the local resolution of cryo-EM density maps. Nat Methods 11:63–65. doi:10.1038/nmeth.2727 24213166 PMC3903095

[63] Emsley P, Lohkamp B, Scott WG, Cowtan K. 2010. Features and development of *Coot*. Acta Crystallogr D Biol Crystallogr 66:486–501. doi:10.1107/S0907444910007493 20383002 PMC2852313

[64] Adams PD, Afonine PV, Bunkóczi G, Chen VB, Davis IW, Echols N, Headd JJ, Hung L-W, Kapral GJ, Grosse-Kunstleve RW, McCoy AJ, Moriarty NW, Oeffner R, Read RJ, Richardson DC, Richardson JS, Terwilliger TC, Zwart PH. 2010. *PHENIX*: a comprehensive Python-based system for macromolecular structure solution. Acta Crystallogr D Biol Crystallogr 66:213–221. doi:10.1107/S0907444909052925 20124702 PMC2815670

[65] Pettersen EF, Goddard TD, Huang CC, Meng EC, Couch GS, Croll TI, Morris JH, Ferrin TE. 2021. UCSF ChimeraX: structure visualization for researchers, educators, and developers. Protein Sci 30:70–82. doi:10.1002/pro.3943 32881101 PMC7737788

