# Supplementary material for: Structure and mechanism of the HSV-1 origin-binding protein UL9
Source: J Virol. 2026 Jul 10;100(8):e00271-26. doi: 10.1128/jvi.00271-26 (PMC13483431; doi:10.1128/jvi.00271-26)
Supplement: Supplementary file 1 [file jvi.00271-26-s0001.pdf]

Supplemental figures

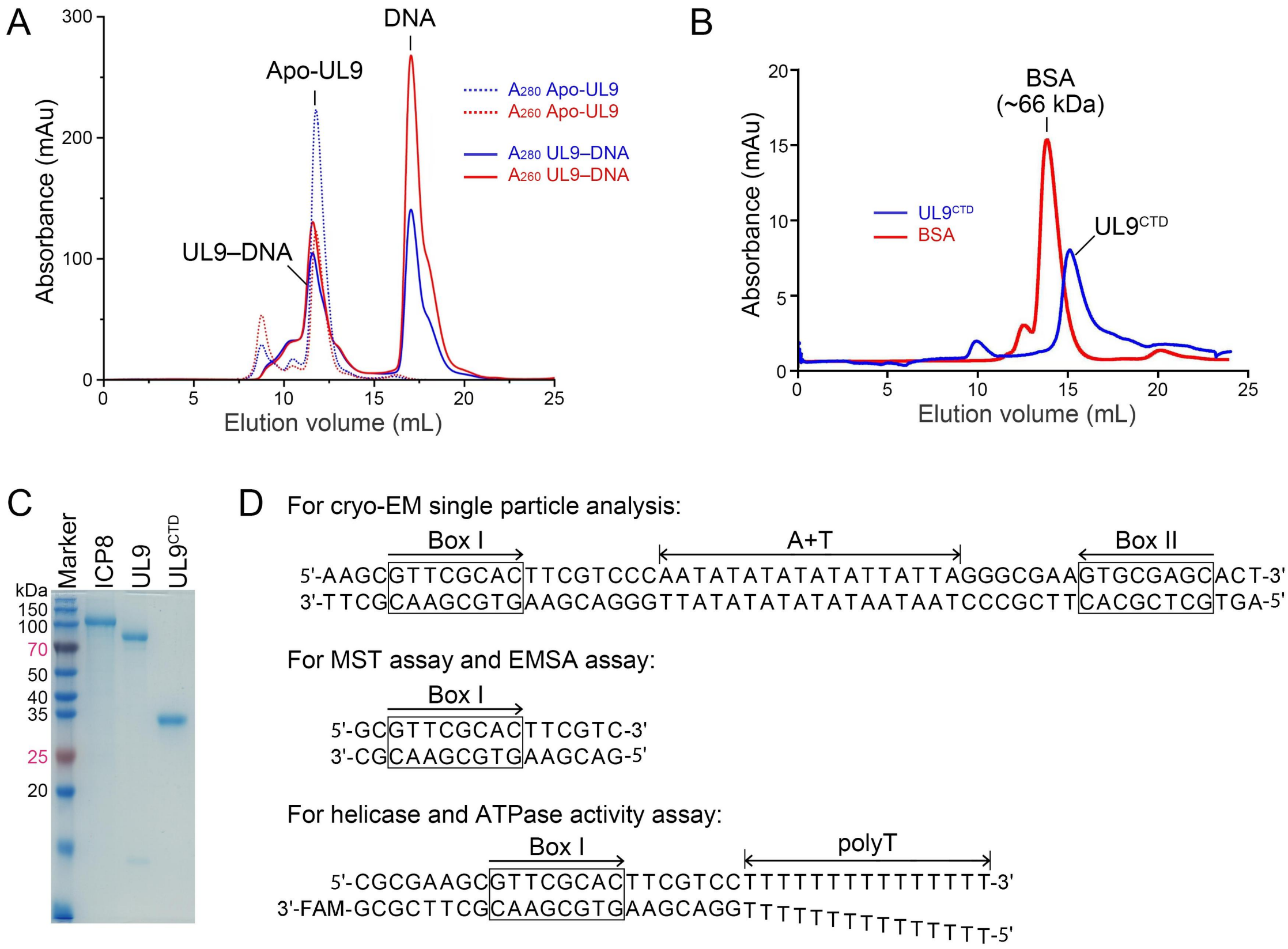

Fig S1 Preparation and characterization of recombinant HSV-1 full-length apo-UL9, UL9–DNA complex, UL9^{CTD}, and ICP8 proteins.

(**A**) Size-exclusion chromatography elution profiles of apo-UL9 (dashed lines) and UL9–DNA complex (solid lines) using a Superdex 200 Increase 10/300 GL column. Ultraviolet absorbance at 280 nm (A_{280}) and 260 nm (A_{260}) indicated in blue and red, respectively. Apo-UL9 predominantly elutes as a single peak corresponding to a stable homodimer (theoretical molecular weight: ~190 kDa). In contrast, the UL9–DNA mixture resolves into two distinct peaks. The first peak elutes earlier than apo-UL9 with an A_{260}/A_{280} ratio of approximately 1.2, representing the successfully assembled UL9–DNA complex (~220 kDa) with a larger hydrodynamic radius. The second peak elutes at approximately 17 mL with an A_{260}/A_{280} ratio of ~2.0, characteristic of the excess, free duplex DNA. (**B**) Size-exclusion chromatography elution profiles of purified UL9^{CTD} (blue line) and bovine serum albumin (BSA, red line) as a molecular weight standard, monitored by ultraviolet absorbance. BSA (~66 kDa) elutes as a sharp peak at approximately 13.8 mL. In comparison, UL9^{CTD} (~32 kDa) elutes at a later volume of approximately 15.1 mL, consistent with its smaller theoretical molecular weight and a monomeric state in solution. (**C**) Coomassie-stained SDS-PAGE analysis of purified UL9^{FL}, UL9^{CTD}, and ICP8 proteins. Molecular weights (in kilodaltons) of the marker are shown on the left, and protein bands are labeled above. Data are representative of at least three independent experiments. (**D**) Sequence and schematic diagrams of the duplex DNA substrates engineered for specific functional and structural characterization. For cryo-EM single-particle analysis, a 56-bp *oriS*-derived duplex substrate containing both the *Box I* and *Box II* binding elements separated by an A+T-rich spacer was utilized. For MST and EMSA assays, a 15-bp duplex substrate containing only the *Box I* element was used. For helicase and ATPase activity assays, a fork duplex substrate containing the *Box I* duplex with a 3′ polyT overhang and a 3′-FAM label on one strand was designed to facilitate binding affinity and gel shift measurements. The conserved origin recognition sequences (*Box I* and *Box II*) are indicated by open boxes with directional arrows.

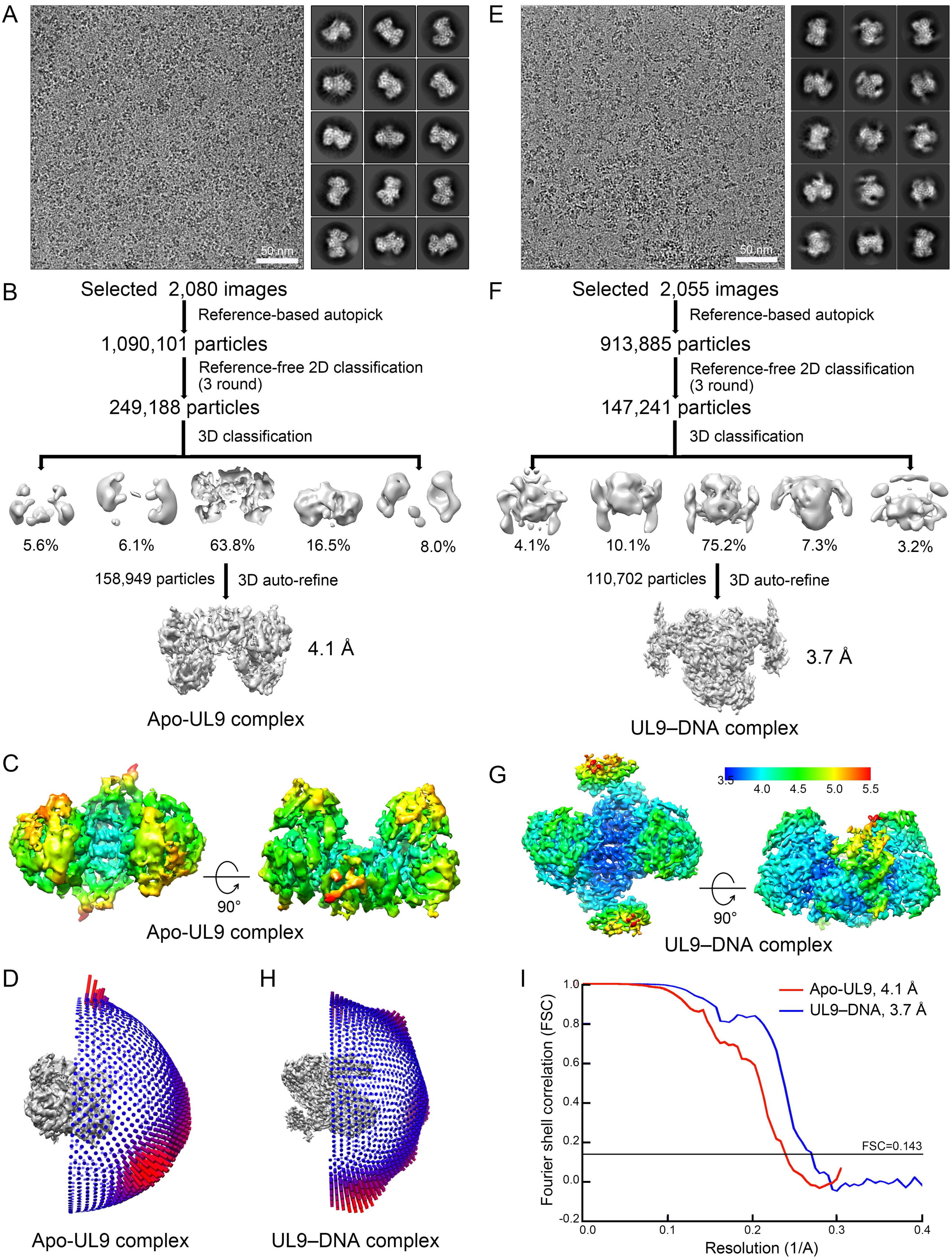

Fig S2 Single-particle cryo-EM analysis of HSV-1 apo-UL9 and UL9–DNA complex.
(**A** and **E**) Representative cryo-EM image and reference-free 2D-class averages of HSV-1 apo-UL9 complex (A) and UL9–DNA complex (E). (**B** and **F**) Data-processing workflow for apo-UL9 complex (B) and UL9–DNA complex (F). (**C** and **G**) Local resolution maps for apo-UL9 complex (C) and UL9–DNA complex (G) estimated by ResMap(63). (**D** and **H**) Angular distribution of the particles of apo-UL9 complex (D) and UL9–DNA complex (H) in the final 3D auto-refinement. (**I**) The gold standard Fourier shell correlation (FSC) curves for the reconstruction of apo-UL9 complex (red line) and UL9–DNA complex (blue line) with resolutions at FSC = 0.143.

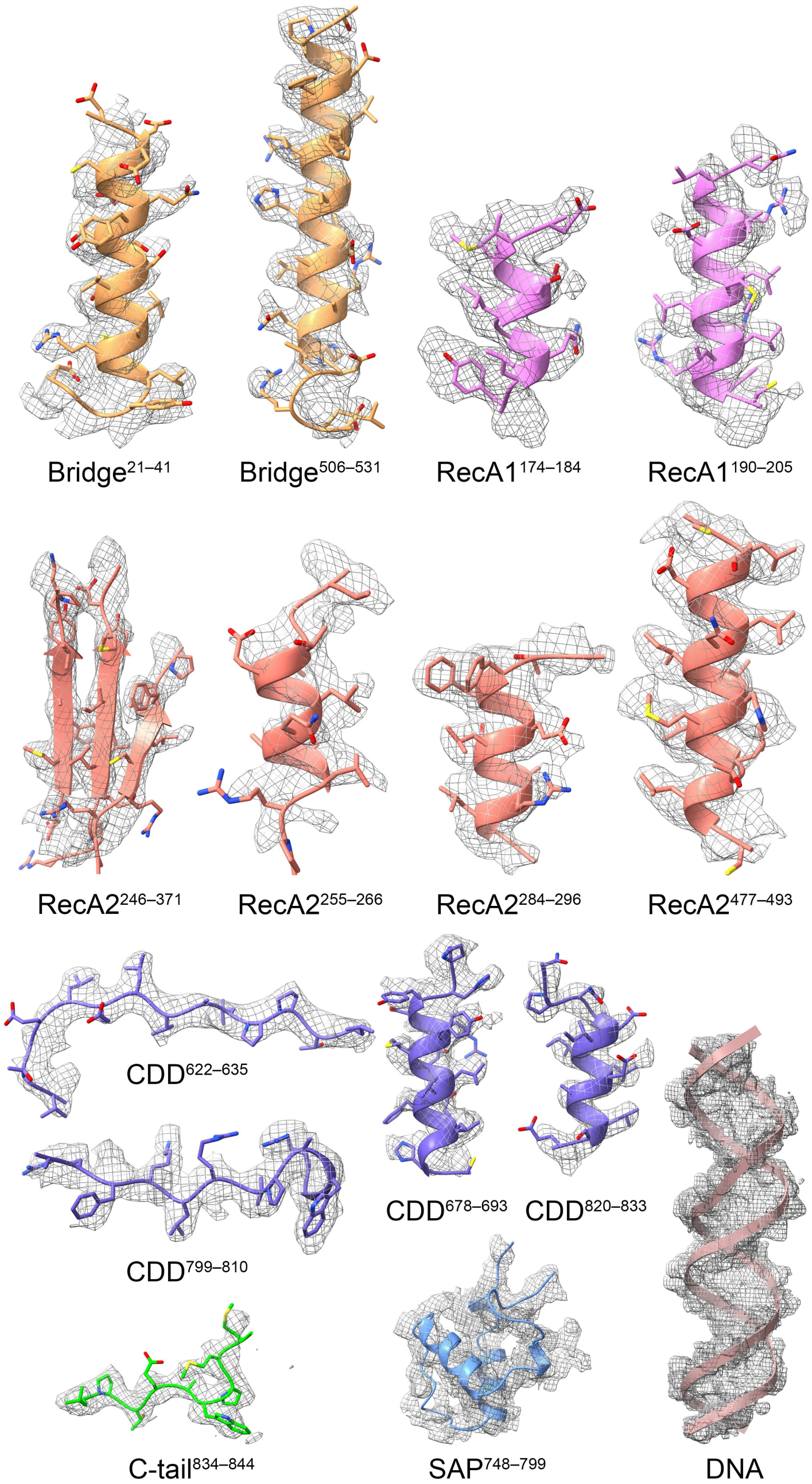

Fig S3 Representative density maps and corresponding atomic models of the UL9–DNA complex. The densities are shown as gray meshes, while the corresponding models are shown as cartoons colored by domain as in Fig. 1A, with side chains displayed as sticks.

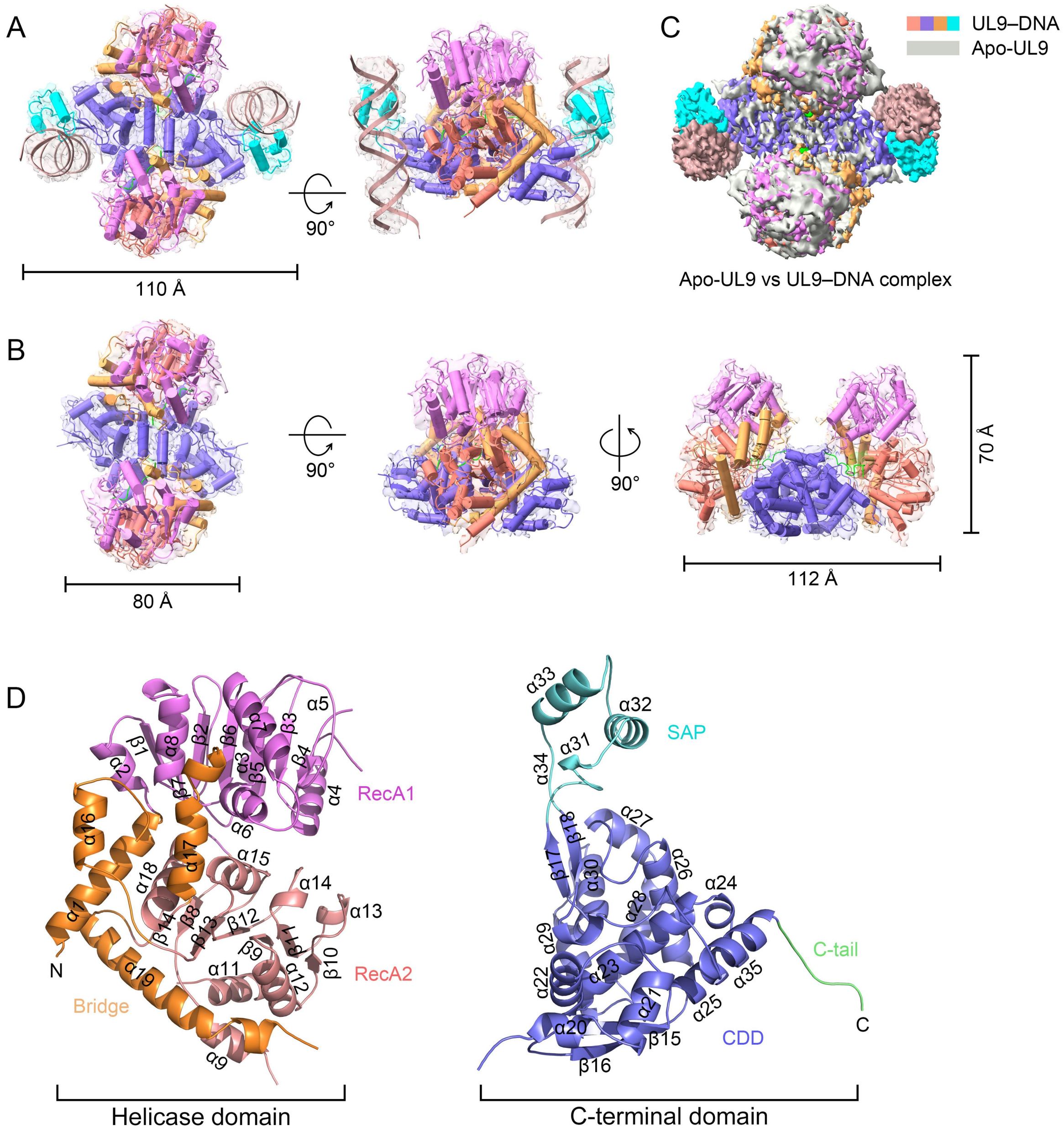

Fig S4 Structural comparison of the apo-UL9 and UL9–DNA complexes.
(**A, B**) Two orthogonal views of the UL9–DNA complex (A) and three orthogonal views of the apo-UL9 homodimer (B), arranged vertically to facilitate direct comparison of their global conformations and domain architectures. The cryo-EM density maps are displayed as transparent gray surfaces, with the atomic models represented as cartoons colored by domain as in Fig. 1A. Measured overall dimensions are labeled in angstroms (Å). (**C**) Superposition of cryo-EM density maps of the apo-UL9 and UL9–DNA complexes. The UL9–DNA complex is colored by domain as in Fig. 1A, while the apo-UL9 map is colored gray. The vertical comparison and structural superposition indicate that the overall architecture of the UL9 dimer remains largely unchanged upon DNA binding. Conformational adjustments are restricted to the SAP subdomain, which undergoes a transition from an intrinsically flexible state to a well-ordered conformation to grip the origin DNA. (**D**) Cartoon representation of the UL9 helicase domain (left) and C-terminal domain (right) with all secondary structure elements explicitly labeled. Zoomed-in views correspond to the regions highlighted in Fig. 2A and B.

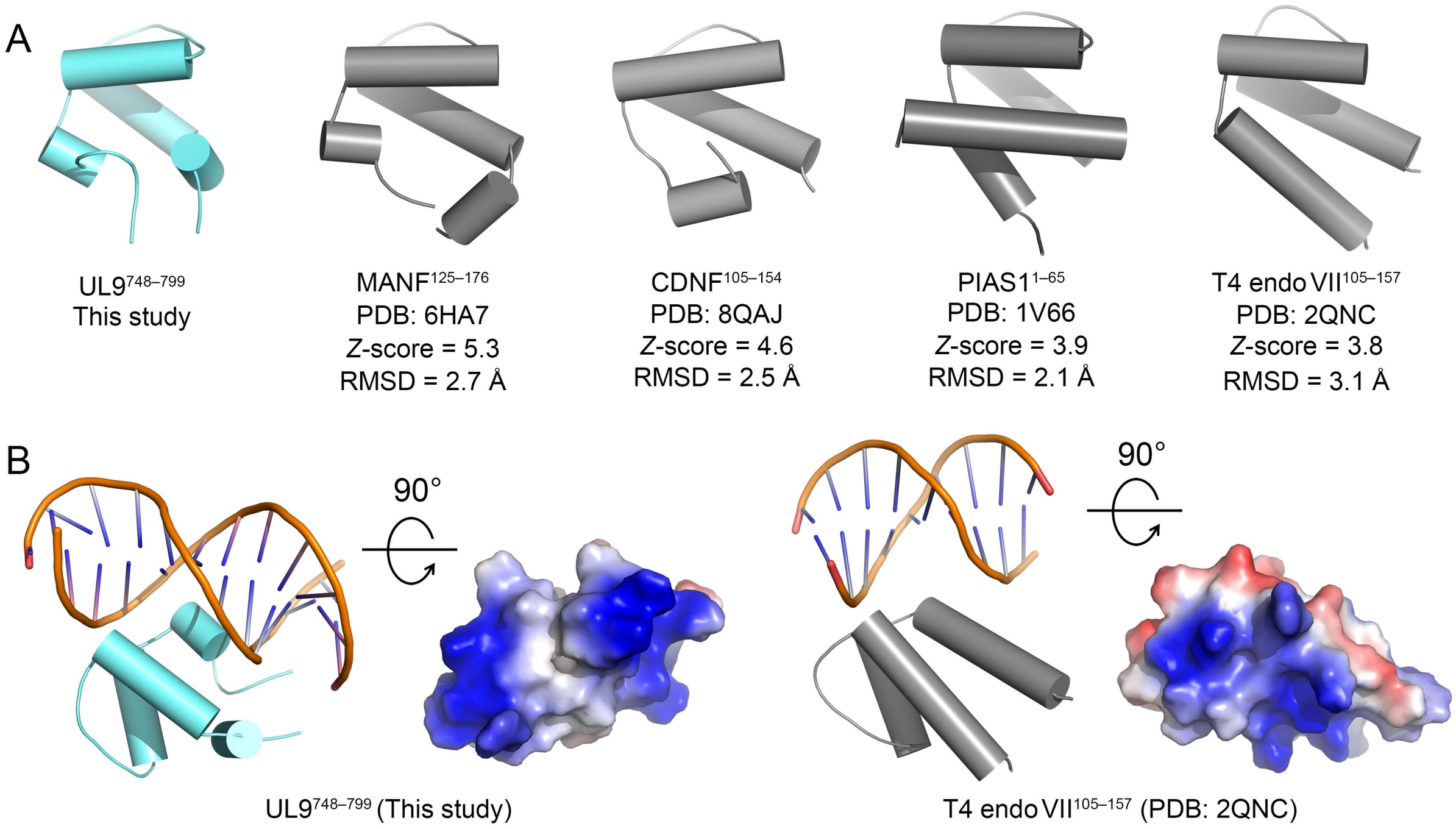

Fig S5 Structural similarity analysis of the SAP subdomain of UL9.
(**A**) Comparison of structurally similar proteins identified through the Dali server. The UL9 SAP subdomain is colored identically to in Fig. 1A, with the structural counterparts displayed in gray. Information regarding these structural analogs, including PDB, residue range, *Z*-score, and RMSD is summarized below. (**B**) Comparison of DNA-binding features (DNA-binding modes and surface electrostatic potential) between the UL9 SAP subdomain and its structure counterpart (T4 endo VII).

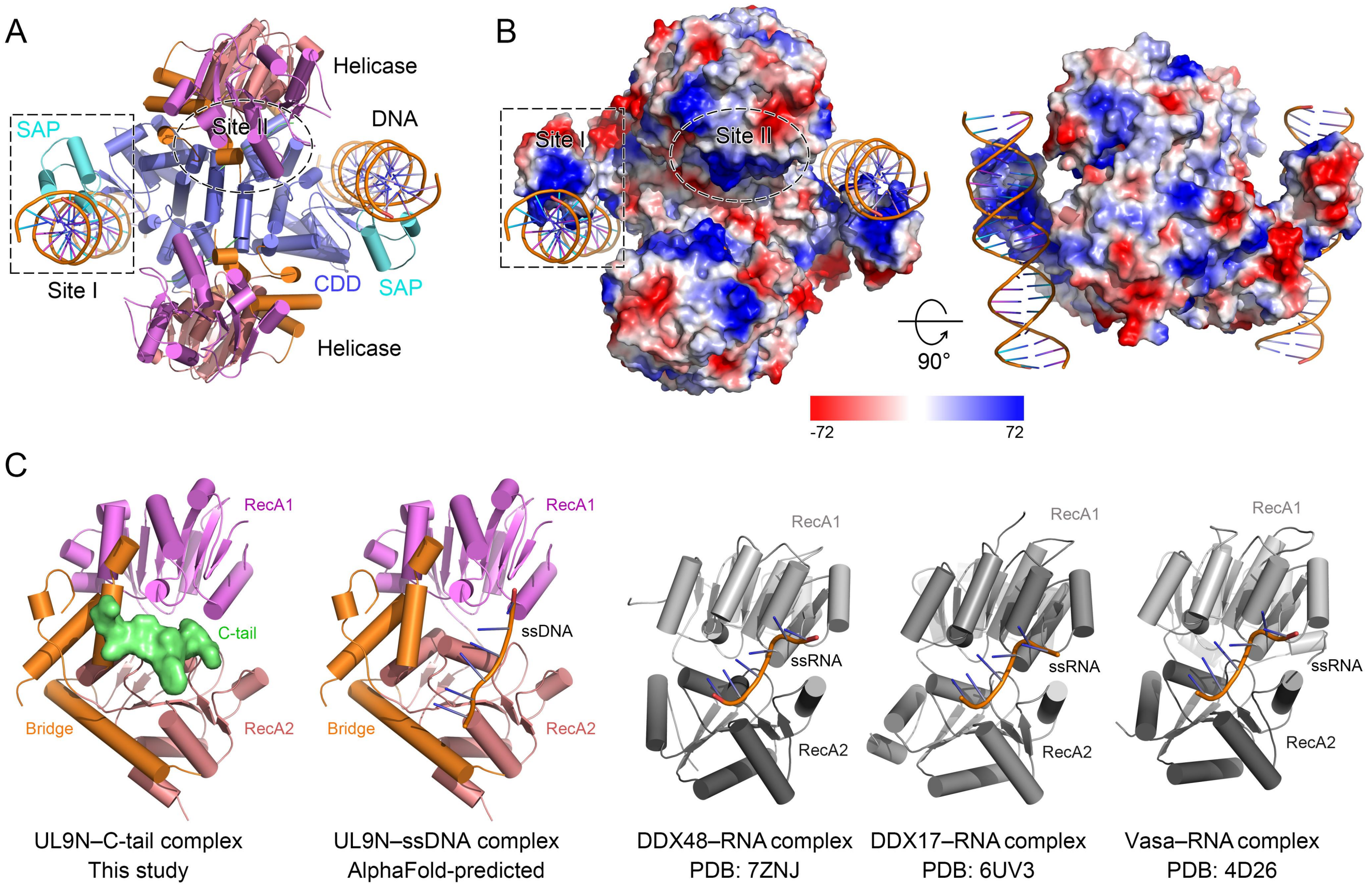

Fig S6 DNA binding properties of HSV-1 UL9.
(**A**) Structural characterization of UL9–DNA interactions shown in cartoon representation. DNA-binding Site **I** was identified in the cryo-EM structure of UL9–DNA complex. DNA-binding Site **II** represents a putative secondary DNA-binding region located on the helicase domain, but no direct helicase–DNA contacts were observed. (**B**) Two orthogonal views of surface electrostatic potential of the dimeric UL9 complex, with scale bar displayed below. (**C**) Structural comparison of UL9 helicase domain binding to the C-tail with a predicted model of the UL9 helicase domain bound to ssDNA, generated by AlphaFold and previous reported helicase-RNA complexes (DDX48–RNA, DDX17–RNA, Vasa–RNA). In panels A and B, the solved Site **I** and predicted Site **II** are marked using dashed rectangles and ellipses, respectively.

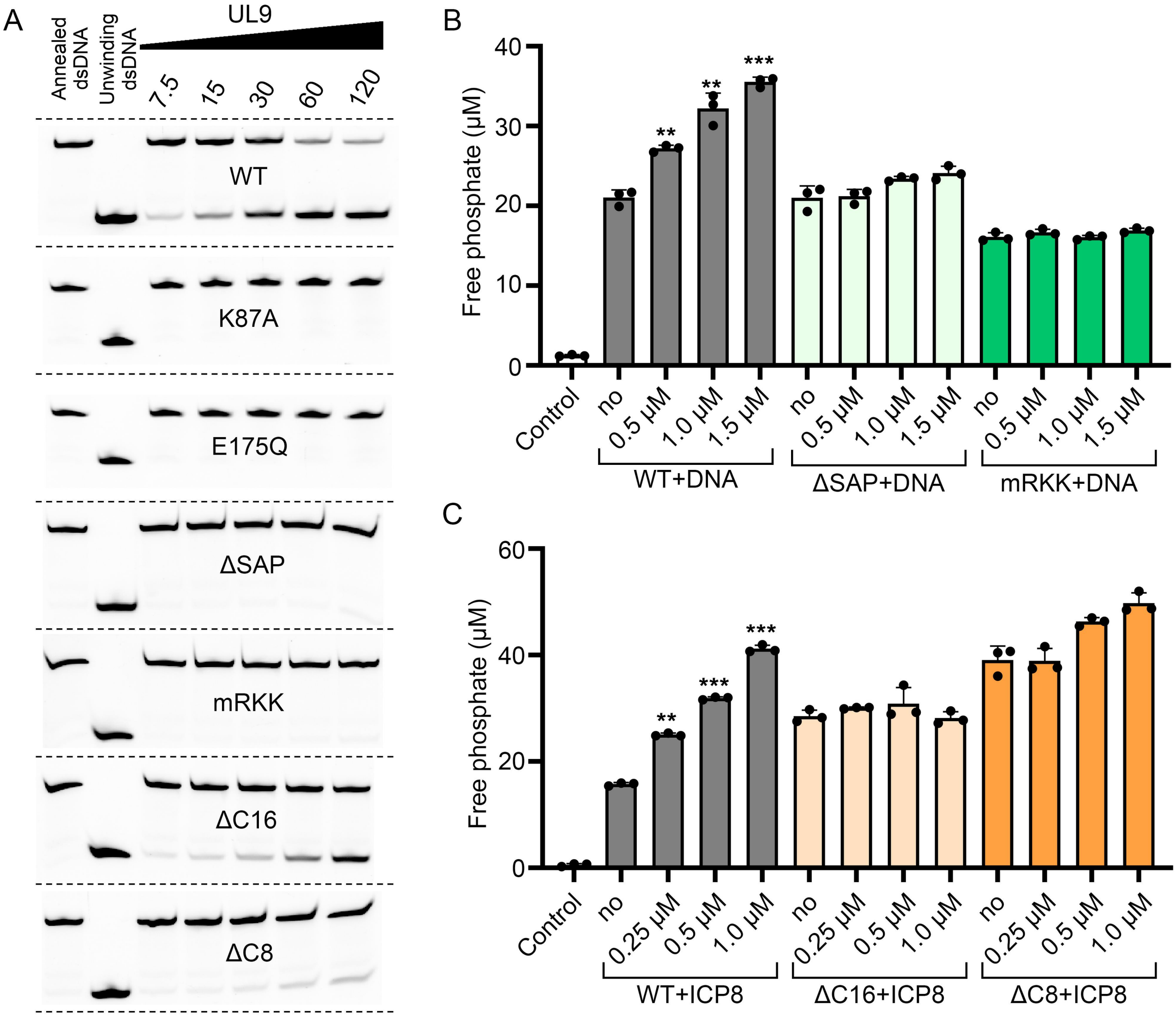

Fig S7 Comparative analysis of helicase and ATPase activities in wild-type UL9 and its mutants.
(**A**) Helicase activity of wild-type UL9 versus its functional mutants. The functional mutants include ATPase-deficient mutants (K87A and E175Q), DNA-binding-deficient mutants (SAP deletion ΔSAP and triple mutation mRKK), and ICP8-binding-defective C-terminal truncations (ΔC16 and ΔC8). Five different protein concentrations were used, ranging from 7.5 to 120 μM. (**B**) Comparative analysis of DNA-stimulated ATPase activity in wild-type UL9 versus DNA-binding-deficient mutants (ΔSAP and mRKK). (**C**) Comparative analysis of ICP8-mediated ATPase activity in wild-type UL9 versus C-terminal truncation mutants (ΔC16 and ΔC8). All assays were repeated independently at least three times with consistent results. Data represent mean ± s.d. of three independent replicates. P values were calculated by a two-tailed Student's *t*-test (*$p < 0.05$, **$p < 0.01$, ***$p < 0.001$).

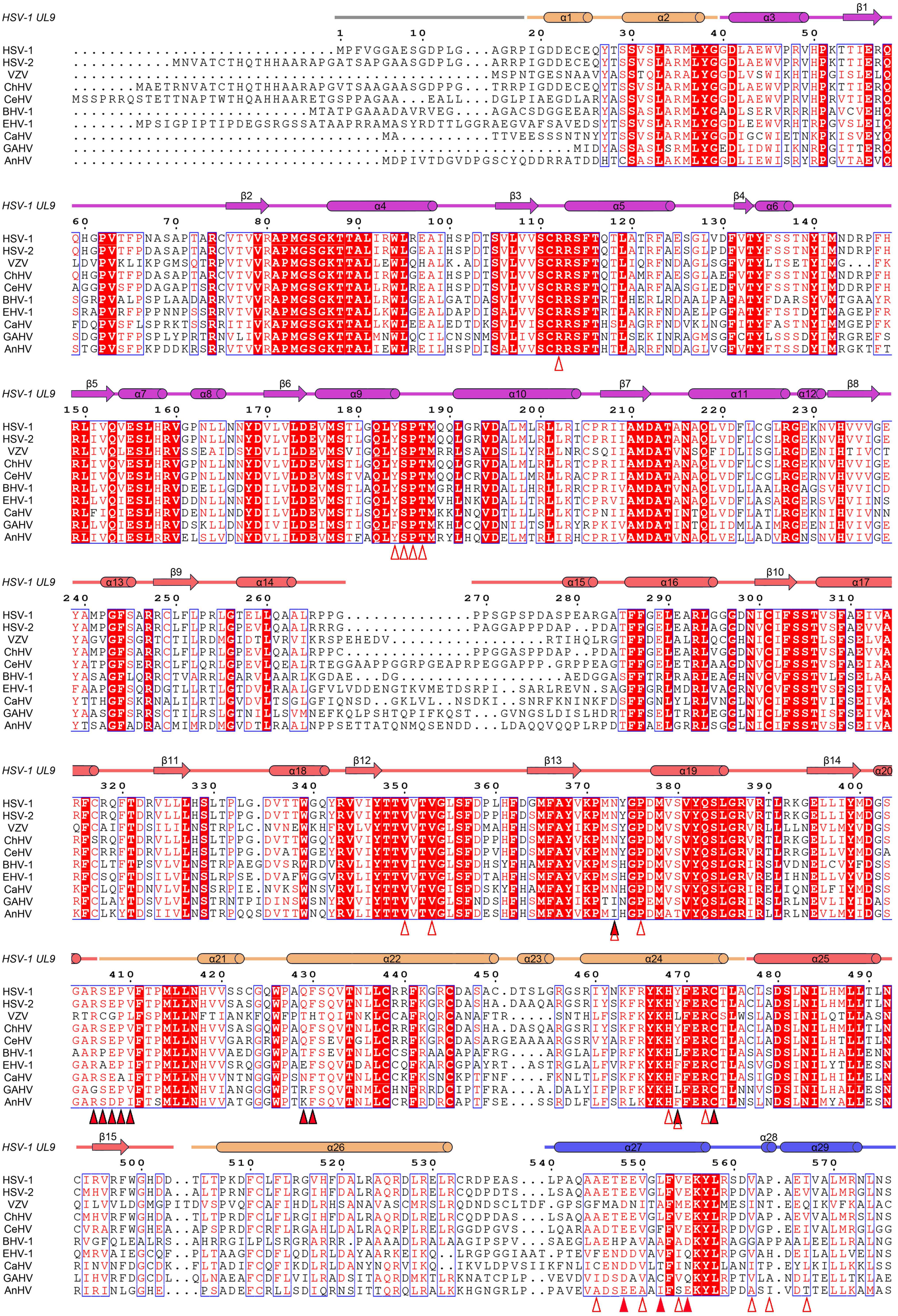

(Figure continued on next page)

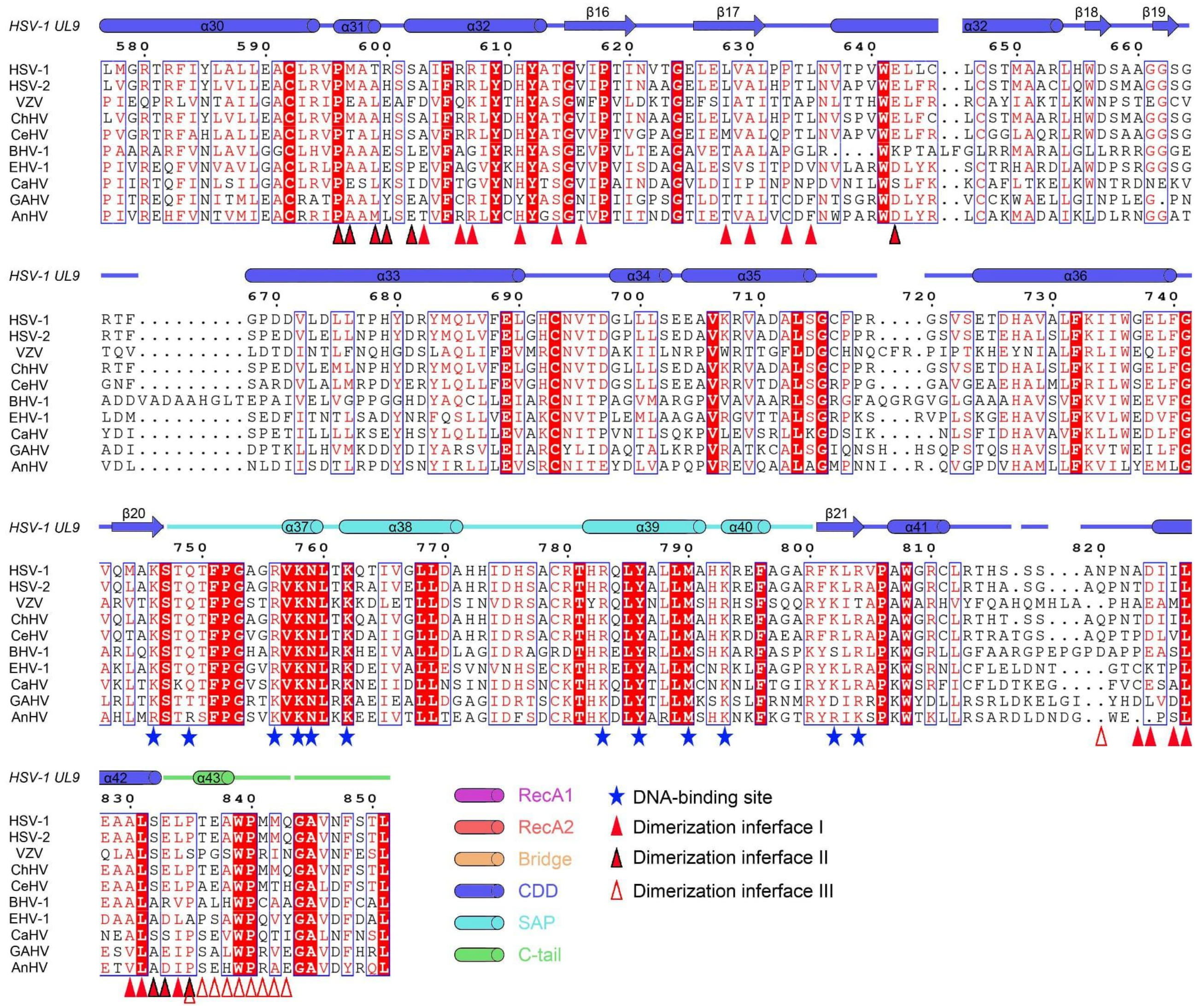

Fig S8 Multiple sequence alignment of representative α-herpesvirus UL9 homologs.

Uniprot accession numbers: HSV-1 (P10193), HSV-2 (P89432), VZV (Q9J3N7), ChHV (K9MG07), CeHV (X2FKV2), BHV-1 (P52377), EHV-1 (P84403), CaHV (A0A172ESJ6), GAHV-1 (Q9E6Q7), AnHV (A0A0U1YYV0). The structure-guided sequence alignment was generated using Clustal Omega(S1) and rendered with ESPript(S2). Completely conserved residues are displayed as white text on red background, while similar residues are shown as red text on white background. Functionally important residues are annotated below the alignment. The solid red triangles, red triangles with a black outline, and hollow red triangles represent dimerization interfaces **I**, **II**, and **III**, respectively, and blue asterisks denote DNA-binding residues. Secondary structure elements corresponding to the solved HSV-1 UL9 structure are depicted above the alignment, colored as in Fig. 1A.

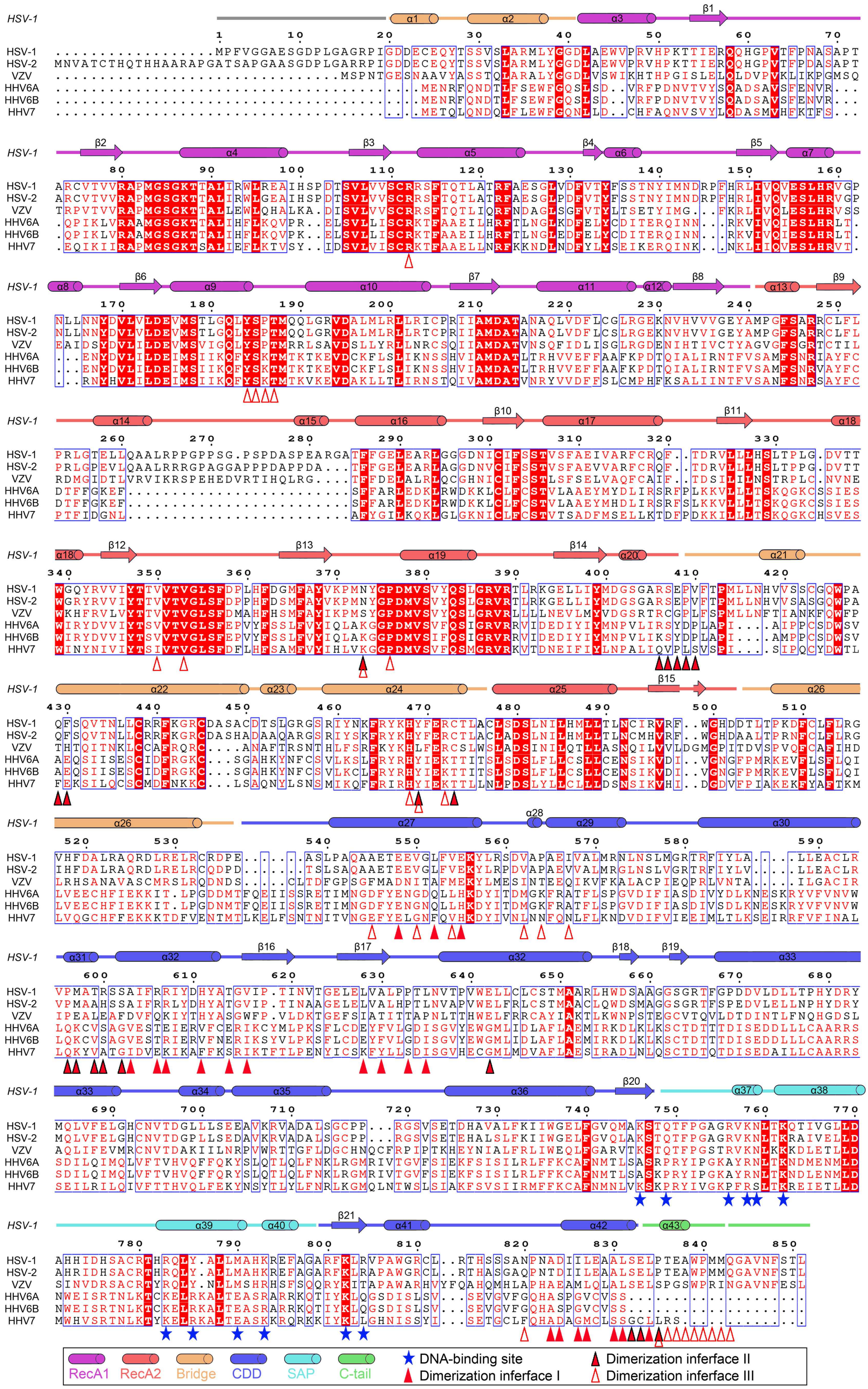

Fig S9 Multiple sequence alignment of the UL9-like proteins encoded by herpesviruses that infect human beings.

Uniprot accession numbers: HSV-1 (P10193), HSV-2 (P89432), VZV (Q9J3N7), HHV6A (P52378), HHV6B (P52452), and HHV7 (P52379). The generation of the sequence alignment and annotation of the results were performed as described for Fig. S8.

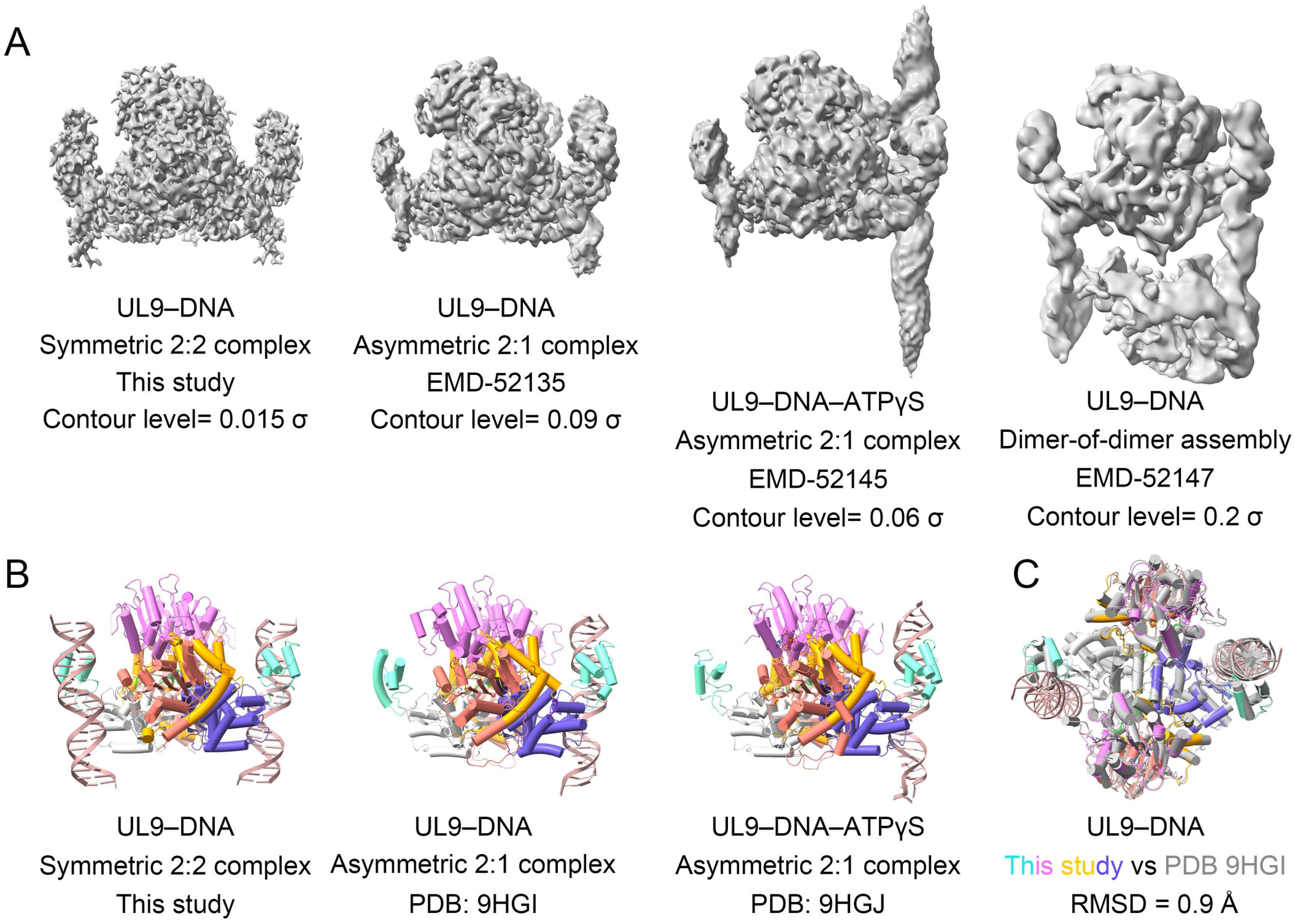

Fig S10 Structural comparison of UL9–DNA complexes.
(**A**) Comparison of cryo-EM density maps. The density map from this study is shown alongside those reported by Gustavsson *et al*.(57), including the dimeric assemblies in the presence and absence of ATPγS, and the tetrameric (dimer–dimer) assembly. EMD accession numbers and contour levels are indicated below each map. (**B**) Cartoon representations of UL9–DNA complexes. The structure determined here is displayed together with the dimeric structures reported by Gustavsson *et al*. in the presence and absence of ATPγS. Corresponding PDB accession codes are listed below each model. (**C**) Superposition of the UL9–DNA complex from this study onto the 2:1 UL9–DNA complex reported by Gustavsson *et al*. The Cα RMSD of 0.9 Å indicates near-identical overall architectures.

Supplemental tables

Table S1. Cryo-EM data collection and validation statistics.

	**Apo-UL9**<br>EMDB-65114<br>PDB: 9VJI	**UL9–DNA complex**<br>EMD-65113<br>PDB: 9VJH
**Data collection and processing**		
Microscope	FEI Titan Krios	FEI Titan Krios
Detector	Gatan K2 Summit	Gatan K2 Summit
Magnification (nominal/calibrated)	165,000	165,000
Voltage (kV)	300	300
Electron exposure ($e^-/Å^2$)	60	60
Defocus range (μm)	–0.8 ~ –3.0	–0.8 ~ –3.0
Pixel size (Å)	0.82	0.82
Symmetry imposed	C_2	C_2
Initial particle images (no.)	955,358	619,808
Final particle images (no.)	158,949	110,702
Map resolution (Å)	4.1	3.7
FSC threshold	0.143	0.143
Map resolution range (Å)	4.0–7.5	3.5–7.5
**Model refinement and validation**		
Initial model used	UL9–DNA complex	AlphaFold-predicted
Model resolution (Å)	4.1	3.7
Model resolution range (Å)	∞ ~ 4.1	∞ ~ 3.7
**Model composition**		
Non-hydrogen atoms	11226	13958
Protein residues	1432	1540
Ligands	0	4
***B* factors ($Å^2$)**		
Protein	47.34	49.41
Ligand	-	131.11
**R.m.s. deviations**		
Bond lengths (Å)	0.004	0.002
Bond angles (°)	0.934	0.523
**Validation**		
MolProbity score	1.62	1.63
Clash score	8.45	8.19
Rotamer outliers (%)	0.00	0.00
**Ramachandran plot**		
Favored (%)	97.09	96.91
Allowed (%)	2.91	3.09
Disallowed (%)	0.00	0.00

Table S2. Structural similar proteins to the UL9 helicase and SAP domain identified by the Dali server.

PDB*	*Z*-score	RMSD (Å)	Protein	Source / Subfamily	Ref
Helicase domain**					
7ZNJ	18.9	3.8	DDX48	DExD/H-box SF2 helicase	(37)
6UV3	18.7	3.9	DDX17	DExD/H-box SF2 helicase	(38)
4D26	15.9	3.9	Vasa	DExD/H-box SF2 helicase	(39)
SAP subdomain**					
6HA7	5.3	2.7	MANF	SAP domain	(40)
8QAJ	4.6	2.5	CDNF	SAP domain	(41)
1V66	3.9	2.1	PIAS1	SAP domain	(42)

Note:

* The top three structural matches with the highest similarity are listed for each region.

** Significant structural counterparts were detected only for the N-terminal helicase domain and the C-terminal SAP subdomain; no structural equivalents were identified for the C-terminal dimerization domain (CDD).

Table S3. Residues and interactions at the UL9 dimerization interface.

Protomer A	Residues	Interaction Types	Residues	Protomer B
**Type I Interface**				
CDD	L552, A603, R606, H611, V616, L628, A630, P633, L635, A823, I826, L827, A830, L831, L834, P835	Van der Waals	L552, A603, R606, H611, V616, L628, A630, P633, L635, A823, I826, L827, A830, L831, L834, P835	CDD
	E548, E555, D824	Salt bridges / Hydrogen bonds	A603, R607	
**Type II Interface**				
CDD	P596, M597	Van der Waals	V410, F430, Y469, C473	Bridge
	T599, S602, E642, S832, E833	Salt bridges / Hydrogen bonds	N373	RecA2
			R406, S407, E408, P409, Q429, Y469	Bridge
**Type III Interface**				
C-tail	P835, A838, W839, P840, M841, M842	Van der Waals	V350, V353, P376	RecA2
			H468, Y469	Bridge
			A545, V550, V554, V562, P564, I567	CDD
	T836, E837, A838, Q843	Salt bridges / Hydrogen bonds	R112, Y184, S185, T187	RecA1
			N373	RecA2
			R472	Bridge
			N820	CDD

Supplemental references

S1. Sievers F, Wilm A, Dineen D, Gibson TJ, Karplus K, Li W, Lopez R, McWilliam H, Remmert M, Soding J, Thompson JD, Higgins DG. 2011. Fast, scalable generation of high-quality protein multiple sequence alignments using Clustal Omega. Mol Syst Biol 7:539. https://doi.org/10. 1038/msb.2011.75

S2. Robert X, Gouet P. 2014. Deciphering key features in protein structures with the new ENDscript server. Nucleic Acids Res 42:W320–4. https://doi.org/10.1093/nar/gku316
